# Synthesis, Characterisation, and *In Vitro* Anticancer Activity of Curcumin Analogues Bearing Pyrazole/Pyrimidine Ring Targeting EGFR Tyrosine Kinase

**DOI:** 10.1155/2013/239354

**Published:** 2013-09-09

**Authors:** Mohamed Jawed Ahsan, Habibullah Khalilullah, Sabina Yasmin, Surender Singh Jadav, Jeyabalan Govindasamy

**Affiliations:** ^1^Department of Pharmaceutical Chemistry, Maharishi Arvind College of Pharmacy, Ambabari, Jaipur, Rajasthan 302 023, India; ^2^Department of Pharmaceutical Chemistry, Alwar Pharmacy College, Alwar, Rajasthan 301 030, India; ^3^Department of Pharmaceutical Chemistry, Birla Institute of Technology, Mesra, Ranchi, Jharkhand 835 215, India

## Abstract

In search of potential therapeutics for cancer, we described herein the synthesis, characterization, and *in vitro* anticancer activity of a novel series of curcumin analogues. The anticancer effects were evaluated on a panel of 60 cell lines, according to the National Cancer Institute (NCI) screening protocol. There were 10 tested compounds among 14 synthesized compounds, which showed potent anticancer activity in both one-dose and 5-dose assays. The most active compound of the series was 3,5-bis(4-hydroxy-3-methylstyryl)-1*H*-pyrazole-1-yl(phenyl)methanone (**10**) which showed mean growth percent of −28.71 in one-dose assay and GI_50_ values between 0.0079 and 1.86 *µ*M in 5-dose assay.

## 1. Introduction

Cancer is still continuing to be a major health problem worldwide. The development of new anticancer therapeutic agents is one of the fundamental goals in medicinal chemistry as cancer causes about 13% of all the death [1]. Surpassing cardiovascular diseases, it is taking the position number one killer due to various factors [2]. Also the treatment of cancer is associated with various side effects which include bone marrow depression, alopecia, drug-induced cancer, hepatotoxicity, and many more. Because of the need and value of anticancer drugs, many laboratories are intensively investigating the chemistry and biology of novel anticancer agents. Also the development of resistance against the existing anticancer drugs and cytotoxicity and genotoxicity of anticancer drugs to the normal cells are other major problems in cancer therapy, keeping research window open in search for newer anticancer molecules [3]. But the window passage has become narrower because it is rather hard to search a molecule that can selectively inhibit the proliferation of abnormal cells only with least or no affect on normal cells.

In the last decade, several pyrazole derivatives proved to have anticancer activity [4–8]. The other activities reported for pyrazole nucleus include antitubercular, anticonvulsant, anticancer, antimicrobial, anti-HIV, antihepatotoxic, anti-inflammatory, and analgesic [9–16]. Also curcumin, a major yellow pigment and active component of turmeric, has been shown to possess anti-inflammatory and anticancer activities [17]. In some countries, curcumin was consumed in the diet up to 4 g per adult/day, which appeared to lower the incidence rate of colorectal cancer. In a study, curcumin showed autophagic and apoptotic death of K562 cell line (leukemia) [18]. The cytotoxicity studies in different cell lines indicated that the toxicity of curcumin was significantly higher in tumor cells if compared to the normal cells [19]. Curcumin and its derivatives possess a wide variety of pharmacological activities, namely, antibacterial, anti-HIV, anti-inflammatory, antimalarial, anticancer, and many more [20–24]. Considering these recent discoveries, curcumin can be considered as an ideal lead compound for anticancer drug development. Earlier we have reported the anticancer activity of pyrazoline and oxadiazole analogues [25, 26]. Receptor tyrosine kinases (RTKs) have been shown to be key regulators of normal cellular processes and to additionally play a critical role in the development and progression of many types of cancer by binding either with polypeptide growth factors or cytokines or hormones [27]. Several transforming oncogenes, like *src*, a gene obtained from Rous sarcoma virus, *abl*, a gene obtained from Abelson murine leukemia virus, and so forth, are known to possess tyrosine kinase activity. Overexpression of certain RTKs shows association with promotion and maintenance of malignancies. Thus, inactivation of the specific tyrosine kinase represents a potential approach for design of anticancer drugs. Gefitinib and erlotinib used in the treatment of certain types of cancer are epidermal growth factor receptor (EGFR) tyrosine kinase inhibitors. Protein tyrosine kinases occupy a central position in the control of cellular proliferation; and it is well recognized that the response of many cells to growth factors is initiated by activation of tyrosine kinase [28]. The involvement of the EGFR family of tyrosine kinases in cancer proliferation suggests that an inhibitor which blocks the tyrosine kinase activity of the entire EGFR family could have significant therapeutic potential [29]. Hence, enthused by all these facts, we have synthesized a novel series of curcumin analogues and evaluated their antitumor activity and selected EGFR family of tyrosine kinase as a biological target for carrying out the docking study of some of the active compounds.

## 2. Materials and Methods

### 2.1. Chemistry

All chemicals were supplied by E. Merck (Germany), Konark Herbal (India), and S. D. Fine Chemicals (India). Melting points were determined by open tube capillary method and are uncorrected. Purity of the compounds was checked by elemental analysis and the progress of reactions was monitored by TLC plates (silica gel G) using mobile phase, hexane : ethylacetate (6 : 4), and the spots were identified by iodine vapours or UV light. IR spectra were obtained on a Shimadzu 8201 PC, FT-IR spectrometer (KBr pellets). ^1^H NMR spectra were recorded on a Bruker AC 300 MHz spectrometer using TMS as internal standard in DMSO. Mass spectra were recorded on a Bruker Esquire LCMS using ESI and elemental analyses were performed on Perkin-Elmer 2400 elemental analyzer.

### 2.2. General Method for the Synthesis of 3,5-Bis(4-hydroxy-3-methylstyryl)-*N*-(substituted phenyl)-1*H*-pyrazole-1-carboxamide Analogues **(1–9)**

1,7-Bis(4-hydroxy-3-methoxyphenyl)hepta-1,6-diene-3,5-dione (curcumin) (0.005 mol) and substituted phenyl semicarbazides (0.005 mol) were refluxed in glacial acetic acid for 12 h. The substituted semicarbazides were synthesized as per reported method [30]. The excess of solvent was removed under reduced pressure, and then the reaction mixture was poured into the crushed ice. The solid mass was filtered, washed, dried, and recrystallized with ethanol furnishing the title 3,5-bis(4-hydroxy-3-methylstyryl)-*N*-(substituted phenyl)-1*H*-pyrazole-1-carboxamide analogues (**1–9**).

#### 2.2.1. 3,5-Bis(4-hydroxy-3-methylstyryl)-*N*-(2-chlorophenyl)-1*H*-pyrazole-1-carboxamide **(1)**

IR: (KBr) cm^−1^: 3381 (OH), 3195 (NH), 1679 (C=O), 1569 (C=N), 1334 (C–N), 745 (C–Cl). ^1^H NMR (300 MHz, DMSO-*d*_6_): *δ* 3.83 (6H, s, OCH_3_), 6.61 (1H, s, CH=C), 6.73 (2H, d, *J *= 15.8 Hz, CH and CH), 6.78 (2H, d, *J* = 15.8 Hz, CH and CH), 6.31–7.62 (10H, m, ArH), 9.72 (2H, s, OH), 9.83 (1H, s, CONH); *m/z* = 517 (M^+^), 519 (M + 2)^+^.

#### 2.2.2. 3,5-Bis(4-hydroxy-3-methylstyryl)-*N*-(4-chlorophenyl)-1*H*-pyrazole-1-carboxamide **(2)**

IR: (KBr) cm^−1^: 3304 (OH), 3192 (NH), 1665 (C=O), 1539 (C=N), 1371 (C–N), 729 (C–Cl). ^1^H NMR (300 MHz, DMSO-*d*_6_): *δ* 3.85 (6H, s, OCH_3_), 6.60 (1H, s, CH=C), 6.77 (2H, d, *J *= 15.8 Hz, CH and CH), 6.81 (2H, d, *J* = 15.8 Hz, CH and CH), 6.91–7.72 (10H, m, ArH), 9.74 (2H, s, OH), 9.93 (1H, s, CONH); ^13^C NMR (75 MHz, DMSO-*d*_6_) ppm: 169.12, 169.03, 148.27, 147.07, 138.60, 138.49, 130.11, 129.04, 128.78, 128.20, 127.04, 120.98, 120.89, 120.51, 115.97, 109.83, 99.74, 55.97; *m/z* = 517 (M^+^), 519 (M + 1)^+^.

#### 2.2.3. 3,5-Bis(4-hydroxy-3-methylstyryl)-*N*-(4-fluorophenyl)-1*H*-pyrazole-1-carboxamide ** (3)**

IR: (KBr) cm^−1^: 3311 (OH), 3181 (NH), 1667 (C=O), 1508 (C=N), 1370 (C–N), 1032 (C–F). ^1^H NMR (300 MHz, DMSO-*d*_6_): *δ* 3.83 (6H, s, OCH_3_), 6.61 (1H, s, CH=C), 6.68 (2H, d, *J *= 16.2 Hz, CH and CH), 6.78 (2H, d, *J *= 16.2 Hz, CH and CH), 6.92–7.60 (10H, m, ArH), 9.62 (2H, s, OH), 9.97 (1H, s, CONH), 12.97 (OH); ^13^C NMR (75 MHz, DMSO-*d*_6_) ppm: 190.1, 158.5, 151.3, 147.3, 147.3, 144.9, 133.4, 123.6, 131.5, 128.8, 123.2, 120.1, 116.8, 115.7, 112.1, 107.7, 56.2; *m/z* = 501 (M^+^), 503 (M + 2)^+^.

#### 2.2.4. 3,5-Bis(4-hydroxy-3-methylstyryl)-*N*-(4-bromophenyl)-1*H*-pyrazole-1-carboxamide **(4)**

IR: (KBr) cm^−1^: 3380 (OH), 3202 (NH), 1689 (C=O), 1569 (C=N), 1330 (C–N), 634 (C–Br). ^1^H NMR (300 MHz, DMSO-*d*_6_): *δ* 3.83 (6H, s, OCH_3_), 6.62 (1H, s, CH=C), 6.67 (2H, d, *J *= 16.6 Hz, CH and CH), 6.71 (2H, d, *J *= 16.6 Hz, CH and CH), 6.92–7.67 (10H, m, ArH), 9.75 (2H, s, OH), 9.97 (1H, s, CONH); *m/z* = 562 (M^+^), 564 (M + 2)^+^.

#### 2.2.5. 3,5-Bis(4-hydroxy-3-methylstyryl)-*N*-(3-chloro-4-fluorophenyl)-1*H*-pyrazole-1-carboxamide ** (5)**

IR: (KBr) cm^−1^: 3305 (OH), 3122 (NH), 1604 (C=O), 1555 (C=N), 1370 (C–N), 737 (C–F). ^1^H NMR (300 MHz, DMSO-*d*_6_): *δ* 3.84 (6H, s, OCH_3_), 6.61 (1H, s, CH=C), 6.75 (2H, d, *J *= 16.2 Hz, CH and CH), 6.78 (2H, d, *J *= 16.2 Hz, CH and CH), 6.90–7.93 (9H, m, ArH), 9.76 (2H, s, OH), 10.14 (1H, s, CONH); ^13^C NMR (75 MHz, DMSO-*d*_6_) ppm: 153.07, 151.95, 141.81, 134.78, 133.53, 132.94, 125.44, 124.33, 122.42, 121.83, 120.78, 114.57, 104.48, 60.74; *m/z* = 535 (M^+^), 537 (M + 1)^+^.

#### 2.2.6. 3,5-Bis(4-hydroxy-3-methylstyryl)-*N*-(4-methylphenyl)-1*H*-pyrazole-1-carboxamide **(6)**

IR: (KBr) cm^−1^: 3384 (OH), 3162 (NH), 1683 (C=O), 1571 (C=N), 1332 (C–N). ^1^H NMR (300 MHz, DMSO-*d*_6_): *δ* 2.09 (3H, s, CH_3_), 3.81 (6H, s, OCH_3_), 6.55 (1H, s, CH=C), 6.63 (2H, d, *J *= 16.8 Hz, CH and CH), 6.68 (2H, d, *J *= 16.8 Hz, CH and CH), 6.82–7.41 (10H, m, ArH), 9.27 (2H, s, OH), 10.02 (1H, s, CONH); *m/z* = 497 (M^+^).

#### 2.2.7. 3,5-Bis(4-hydroxy-3-methylstyryl)-*N*-(2-methylphenyl)-1*H*-pyrazole-1-carboxamide **(7)**

IR: (KBr) cm^−1^: 3361 (OH), 3151 (NH), 1686 (C=O), 1569 (C=N), 1337 (C–N). ^1^H NMR (300 MHz, DMSO-*d*_6_): *δ* 2.12 (3H, s, CH_3_), 3.84 (6H, s, OCH_3_), 6.57 (1H, s, CH=C), 6.64 (2H, d, *J *= 16.8 Hz, CH and CH), 6.69 (2H, d, *J *= 16.8 Hz, CH and CH), 6.89–7.39 (10H, m, ArH), 9.35 (2H, s, OH), 10.23 (1H, s, CONH); *m/z* = 497 (M^+^).

#### 2.2.8. 3,5-Bis(4-hydroxy-3-methylstyryl)-*N*-(2,4-dimethylphenyl)-1*H*-pyrazole-1-carboxamide **(8)**

IR: (KBr) cm^−1^: 3310 (OH), 3104 (NH), 1681 (C=O), 1559 (C=N), 1374 (C–N). ^1^H NMR (300 MHz, DMSO-*d*_6_): *δ* 1.19 (6H, s, CH_3_), 3.83 (6H, s, OCH_3_), 6.52 (1H, s, CH=C), 6.61 (2H, d, *J *= 16.8 Hz, CH and CH), 6.65 (2H, d, *J *= 16.8 Hz, CH and CH), 6.78–7.43 (9H, m, ArH), 9.71 (2H, s, OH), 10.18 (1H, s, CONH); ^13^C NMR (75 MHz, DMSO-*d*_6_) ppm: 168.60, 148.33, 147.22, 134.49, 134.35, 132.0, 131.20, 130.03, 128.77, 128.18, 126.82, 125.54, 120.55, 116.04, 109.84, 99.78, 55.99; *m/z* = 511 (M^+^).

#### 2.2.9. 3,5-Bis(4-hydroxy-3-methylstyryl)-*N*-(2,6-dimethylphenyl)-1*H*-pyrazole-1-carboxamide **(9)**

IR: (KBr) cm^−1^: 3383 (OH), 3164 (NH), 1682 (C=O), 1576 (C=N), 1321 (C–N). 1H NMR (300 MHz, DMSO-*d*_6_): *δ* 1.22 (6H, s, CH_3_), 3.85 (6H, s, OCH_3_), 6.60 (1H, s, CH=C), 6.73 (2H, d, *J *= 6.2 Hz, CH and CH), 6.76 (2H, d, *J *= 6.2 Hz, CH and CH), 6.89–7.92 (9H, m, ArH), 9.97 (2H, s, OH), 10.14 (1H, s, CONH); *m/z *= 511 (M^+^).

### 2.3. General Method for the Synthesis of 3,5-Bis(4-hydroxy-3-methylstyryl)-1*H*-pyrazole-1-yl (substituted phenyl)methanone Analogues **(10**-11**)**

1,7-Bis(4-hydroxy-3-methoxyphenyl)hepta-1,6-diene-3,5-dione (curcumin) (0.005 mol) and substituted phenyl hydrazides (0.005 mol) were refluxed in glacial acetic acid for 12 h. The excess of solvent was removed under reduced pressure, and then the reaction mixture was poured into the crushed ice. The solid mass was filtered, washed, dried, and recrystallized with ethanol furnishing the title 3,5-bis(4-hydroxy-3-methylstyryl)-1*H*-pyrazole-1-yl (substituted phenyl)methanone analogues (**10-11**).

#### 2.3.1. 3,5-Bis(4-hydroxy-3-methoxystyryl)-1*H*-pyrazole-1-yl(phenyl)methanone **(10)**

IR: (KBr) cm^−1^: 3342 (OH), 3168 (NH), 1755 (C=O), 1578 (C=N), 1378 (C–N). ^1^H NMR (300 MHz, DMSO-*d*_6_): *δ* 3.96 (6H, s, OCH_3_), 6.21 (1H, s, CH=C), 6.73 (2H, d, *J *= 15.2 Hz, C_2_H and C_6_H), 6.75 (2H, d, *J *= 15.2 Hz, C_1_H and C_7_H), 6.87–7.57 (11H, m, ArH), 9.92 (2H, s, OH); ^13^C NMR (75 MHz, DMSO-*d*_6_) ppm: 159.06, 156.32, 153.66, 147.90, 146.80, 145.74, 137.69, 137.42, 135.15, 134.24, 129.70, 128.31, 119.52, 115.60, 111.75, 110.28, 55.58; MS: *m/z*, (M^+^) 468.

#### 2.3.2. 3,5-Bis(4-hydroxy-3-methoxystyryl)-1*H*-pyrazole-1-yl(2-bromophenyl)methanone **(11)**

IR: (KBr) cm^−1^: 3339 (OH), 3121 (NH), 1757 (C=O), 1514 (C=N), 1371 (C–N), 596 (C–Br). ^1^H NMR (300 MHz, DMSO-*d*_6_): *δ* 3.98 (6H, s, OCH_3_), 6.20 (1H, s, CH=C), 6.75 (2H, d, *J *= 15.8 Hz, C_2_H and C_6_H), 6.78 (2H, d, *J *= 15.8 Hz, C_1_H and C_7_H), 6.82–7.42 (10H, m, ArH), 9.47 (2H, s, OH); MS: *m/z*, (M^+^) 547, (M + 2)^+^ 549.

### 2.4. General Method for the Synthesis of Dihydropyrimidine Analogues **(12–14)**

1,7-Bis(4-hydroxy-3-methoxyphenyl)hepta-1,6-diene-3,5-dione (curcumin) (0.005 mol) and urea/guanidine/thiourea (0.005 mol) were refluxed in glacial acetic acid for 12 h. The excess of solvent was removed under reduced pressure, and then the reaction mixture was poured into the crushed ice. The solid mass was filtered, washed, dried, and recrystallized with ethanol furnishing the title 3,5-bis(4-hydroxy-3-methylstyryl)-*N*-(substituted phenyl)-1*H*-pyrazole-1-carboxamide analogues (**12–14**).

#### 2.4.1. 4,6-Bis(4-hydroxy-3-methoxystyryl)pyrimidine-2(1H)-one ** (12)**

IR: (KBr) cm^−1^: 3402 (OH), 3192 (NH), 1633 (C=O), 1573 (C=N), 1371 (C–N). ^1^H NMR (300 MHz, DMSO-*d*_6_): *δ* 3.79 (6H, s, OCH_3_), 4.92 (1H, s, NH), 5.1 (1H, s, NH), 6.23 (1H, s, CH=C), 6.61 (2H, d, *J *= 14.2 Hz, C_2_H and C_6_H), 6.65 (2H, d, *J *= 14.2 Hz, C_1_H and C_7_H), 6.69–7.22 (6H, m, ArH), 9.96 (2H, s, OH); MS: *m/z*, (M^+^) 391, (M + 1)^+^ 392.

#### 2.4.2. 4,4′-[(2-Imino-1,2-dihydropyrimidine-4,6-diyl)diethene-2,1-diyl]bis(2-methoxyphenol) **(13)**

IR: (KBr) cm^−1^: 3423 (OH), 3124 (NH), 1583 (C=N), 1375 (C–N). ^1^H NMR (300 MHz, DMSO-*d*_6_): *δ* 3.73 (6H, s, OCH_3_), 4.9 (1H, s, NH), 5.2 (1H, s, NH), 6.21 (1H, s, CH=C), 6.66 (2H, d, *J *= 14.2 Hz, C_2_H and C_6_H), 6.69 (2H, d, *J *= 14.2 Hz, C_1_H and C_7_H), 6.71–7.14 (6H, m, ArH), 9.75 (2H, s, OH); MS: *m/z*, (M^+^) 391, (M + 1)^+^ 392.

#### 2.4.3. 4,6-Bis[2-(4-hydroxy-3-methoxyphenyl)ethenyl]pyrimidine-2(1H)-thione **(14)**

IR: (KBr) cm^−1^: 3419 (OH), 3114 (NH), 1581 (C=N), 1365 (C–N), 1273 (C=S). ^1^H NMR (300 MHz, DMSO-*d*_6_): *δ* 3.74 (6H, s, OCH_3_), 5.1 (1H, s, NH), 6.61 (1H, s, CH=C), 6.65 (2H, d, *J *= 14.6 Hz, C_2_H and C_6_H), 6.68 (2H, d, *J *= 14.6 Hz, C_1_H and C_7_H), 6.78–7.12 (6H, m, ArH), 9.72 (2H, s, OH); MS: *m/z*, (M^+^) 408, (M + 1)^+^ 409.

### 2.5. Anticancer Activity

There were 10 compounds among the series, selected and screened for their anticancer activity both in one-dose and 5-dose assays by National Cancer Institute (NCI) on leukemia, melanoma, lung, colon, CNS, ovarian, renal, prostate, and breast cancers cell lines, nearly 60 in number according to their screening protocol, reported elsewhere [31–34]. All the curcumin analogues were synthesized, and the structure of the compounds was submitted online to the official site of NCI for anticancer screening. Among 14 compounds only 10 compounds were selected for anticancer screening. NCI has its own selection procedure of the compounds for anticancer screening based on the novelty of heterocyclic ring system, drug-like properties utilizing the concept of privileged scaffolds, structure based on computer-aided drug design, and so forth, while the structures containing problematic linkage or functional groups (e.g., nitro, nitroso, –N–N–, –N=N–, imine, semicarbazone, thioamides, and thioureas) for successful drug development are avoided [31].

The anticancer screening was carried out as per the NCI US protocol. Using the seven absorbance measurements (time zero, (*T*_*i*_), control growth, (*C*), and test growth in the presence of drug at the five concentration levels (*T*_*f*_)), the percentage growth was calculated at each of the drug concentrations level as [(*T*_*f*_ − *T*_*i*_)/(*C* − *T*_*i*_)] × 100 for concentrations for which *T*_*f*_ ≥ *T*_*i*_ and [(*T*_*f*_ − *T*_*i*_)/*T*_*i*_] × 100 for concentrations for which *T*_*f*_ < *T*_*i*_.

Three-dose response parameters (GI_50_, TGI, and LC_50_) were calculated for each of the experimental agents. Growth inhibition of 50% (GI_50_) was calculated from 100 × [(*T*_*f*_ − *T*_*i*_)/(*C* − *T*_*i*_)] = 50, which was the drug concentration resulting in a 50% reduction in the net protein increase (as measured by sulforhodamine B, SRB staining) in control cells during the drug incubation. The total growth inhibition (TGI) was calculated from *T*_*f*_ = *T*_*i*_, which was the drug concentration resulting in total growth inhibition and signified the cytostatic effect. The LC_50_ was calculated from 100 × [(*T*_*f*_ − *T*_*i*_)/*T*_*i*_] = −50, indicating a net loss of cells following treatment which indicated the concentration of drug resulting in a 50% reduction in the measured protein at the end of the drug treatment as compared to that at the beginning. Values were calculated for each of these three parameters at the level of activity; however, if the effect did not reach to the level of activity, the value of parameter was expressed as less than the minimum concentration tested, or if the effect exceeded the level of activity, the value of parameter was expressed as greater than the maximum concentration tested [31, 35, 36]. Log GI_50_, log TGI, and log LC_50_ are the logarithm molar concentrations producing 50% growth inhibition (GI_50_), a total growth inhibition (TGI), and a 50% cellular death (LC_50_), respectively.

### 2.6. Molecular Docking Studies

#### 2.6.1. Protein Structure

X-ray crystal structure EGFR kinase (PDB: 2J5F), with resolution 3.00 Å; *R*-value: 0.194 (obs.), was obtained from the protein data bank (Research Collaboratory for Structural Bioinformatics (RCSB) (http://www.rcsb.org/pdb)).

#### 2.6.2. Protein Preparation

The protein (PDB: 2J5F) was prepared using the Protein Preparation Wizard. Preprocessed bond orders were assigned, hydrogens were added, metals were treated, and water molecules were deleted. Heterostate for cocrystallized ligand was generated using Epik; protonation state and optimization of H-bonding of the protein side chains were assigned using ProtAssign. Energy was minimized (Impref minimization) using RMSD 0.30 Å and converged by OPLS2005 force field utilities of Schrödinger's suite 9.3.

#### 2.6.3. Receptor Grid Generation

Receptor Grid has been generated with GLIDE module of Schrödinger with default parameters and without any constraints. Site has been specified as centroid of the work space ligand (20 Å) with van der Waals radius scaling factor 1.0 and partial charge cutoff 0.25.

#### 2.6.4. Molecular Docking Protocol

The ligand docking was performed in GLIDE5.0. Ligands which show less than 35 rotatable bonds and which are having less than 200 atoms were selected. The scaling factor will be 0.80 and the potential charge cutoff is 0.15. All the conformers from the confgen-ligprep output were docked in the EGFR Tyrosine Kinase active site. All default parameters were used for extra precision docking. Glide extra precision mode was employed for the current docking study. Best poses were chosen for energy minimization during docking, a distance dependent dielectric constant of 2.0 and maximum number of minimization step of 100 was used. The docking simulations (Ligand receptor interactions) are scored using the Xtra precision (XP) mode which is implemented in GLIDE5.0.

### 2.7. Materials and Methods

All computational analysis was carried out on a Red Hat 5.0 Linux platform running on a Dell Precision work station with Intel core 2 quad processor and 8 GB of RAM.

## 3. Results and Discussion

### 3.1. Chemistry

**Scheme 1:**
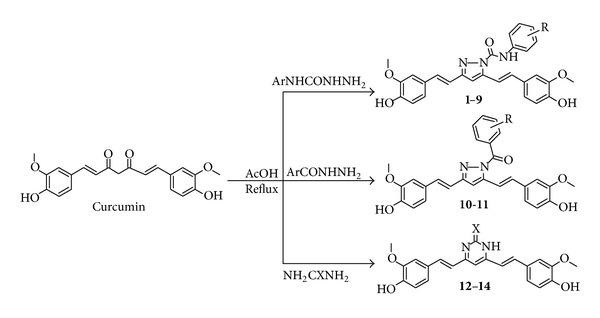
Protocol for the synthesis of curcumin analogues.

**Figure 1:**
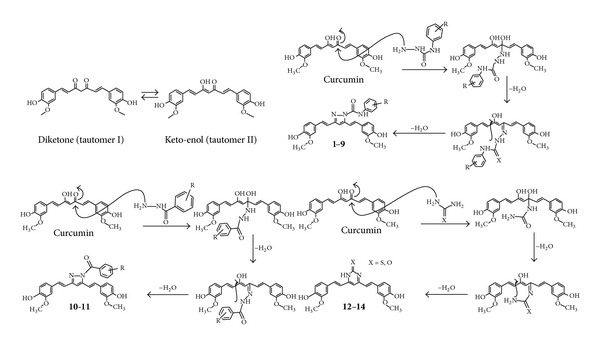
Plausible mechanism of reaction for the synthesis of pyrazole analogues (**1–14**).

The curcumin analogues (**1–14**) described in the study are shown in Table 1, and the reaction sequence for the synthesis is summarized in Scheme 1. In the first part of the synthesis, 1,7-bis(4-hydroxy-3-methoxyphenyl)hepta-1,6-diene-3,5-dione (curcumin) and substituted phenyl semicarbazide were refluxed in glacial acetic acid to obtain the 3,5-bis(4-hydroxy-3-methoxystyryl)-*N*-(substituted phenyl)-1*H*-pyrazole-1-carboxamide analogues (**1–9**). Substituted phenyl semicarbazides (ArNHCONHNH_2_) were synthesized as per reported method [30]. In the second part of the synthesis, 1,7-bis(4-hydroxy-3-methoxyphenyl)hepta-1,6-diene-3,5-dione (curcumin) and substituted phenylhydrazide (ArCONHNH_2_) were refluxed in glacial acetic acid to obtain the 3,5-bis(4-hydroxy-3-methylstyryl)-1*H*-pyrazole-1-yl(substituted phenyl)methanone (**10-11**). In the third part of synthesis, 1,7-bis(4-hydroxy-3-methoxyphenyl)hepta-1,6-diene-3,5-dione (curcumin) and urea/guanidine/thiourea were refluxed in glacial acetic acid to obtain the pyrimidine analogues (**12–14**). The plausible mechanism of reactions is given in Figure 1. The yields of the title compounds were ranging from 66% to 88% after recrystallization with absolute ethanol. The completion of reaction was monitored by TLC using mobile phase, hexane : ethylacetate (6 : 4), and purity of the compounds was checked by elemental analyses. Both the analytical and spectral data (IR, NMR, and MS) of the synthesized compounds were in full accordance with the proposed structures. In general, IR spectra of the compounds afforded C=N stretching at 1514–1583 cm^−1^ and C–N stretch at 1320–1335 cm^−1^ and carbamoyl group N–H stretching at 3122–3210 cm^−1^ and C=O stretching at 1680–1689 cm^−1^ bands for carboxamide analogues (**1–9**) and at 1755–1757 cm^−1^ bands for methanone analogues (**10-11**). The ^1^H NMR spectra showed singlet at *δ* 1.19–1.28 ppm corresponding to CH_3_; a singlet at *δ* 3.79–3.85 ppm corresponding to OCH_3_; a singlet at *δ* 6.52–6.62 ppm corresponding to CH=C proton (pyrazole/dihydropyrimidine); a doublet at *δ* 6.61–6.75 ppm corresponding to CH=CH proton; a doublet at 6.65–6.81 ppm corresponding to CH=CH proton; a multiplet at *δ* 6.81–7.93 ppm corresponding to aromatic protons; broad singlet at *δ* 9.93–10.16 ppm corresponding to CONH_2_. The compounds (**1–14**) in ^1^H NMR spectra exhibited two doublets with *J* value between 14.6 and 16.6 Hz confirming the *trans* coupling. The mass spectra of the compounds revealed in each case, a peak corresponding to their molecular ion peaks. The elemental analysis results were within ±0.4% of the theoretical values.

### 3.2. Anticancer Activity

Ten compounds were evaluated for their anticancer activity in both one-dose and 5-dose assays. The observed anticancer screening data of the compounds are given in Table 2. The 5-dose assay screening data of three compounds are given in Table 3. Compound **1** was found to be highly active on COLO 205 (colon cancer) with cell promotion of −73.49% followed by RXF 393 (renal cancer) with cell promotion of −50.32% and HT29 (colon cancer) with cell promotion of −34.95% while the maximum cell promotion was observed on NCI/ADR-RES (ovarian cancer), which showed 20.83% growth promotion (79.17% growth inhibition) at one-dose assay. Compound **2** was found to be highly active on RXF 393 (renal cancer) with cell promotion of −53.60% followed by SK-MEL-5 (melanoma) with cell promotion of −29.66% and MDA-MB-468 (breast cancer) with cell promotion of −26.40% while the maximum cell promotion was observed on TK-10 (renal cancer), which showed 23.10% growth promotion (76.90% growth inhibition) at one-dose assay. The compound **3** was found to be highly active on RXF 393 (renal cancer) with cell promotion of −43.84% followed by HT29 (colon cancer) with cell promotion of −41.84% and SK-MEL-2 (melanoma) with cell promotion of −27.07% while the maximum cell promotion was observed on TK-10 (renal cancer), which showed 38.12% growth promotion (61.88% growth inhibition) at one-dose assay. Compound **5** was found to be highly active on COLO 205 (colon cancer) with cell promotion of −59.43% followed by RXF 393 (renal cancer) with cell promotion of −57.61% and SF-295 (CNS cancer) with cell promotion of −55.32% while the maximum cell promotion was observed on TK-10 (renal cancer), which showed 24.04% growth promotion (75.96% growth inhibition) at one-dose assay. Compound **6** was found to be highly active on RXF 393 (renal) with cell promotion of −47.46% followed by MDA-MB-468 (breast cancer) with cell promotion of −29.14% and SK-OV-3 (ovarian cancer) with cell promotion of −29.09% while the maximum cell promotion was observed on TK-10 (renal cancer), which showed 28.55% growth promotion (71.45% growth inhibition) at one-dose assay. Compound **8** was found to be highly active on SK-MEL-5 (melanoma) with cell promotion of −83.44% followed by RXF 393 (renal cancer) with cell promotion of −67.29% and SK-OV-3 (ovarian cancer) with cell promotion of −63.58% while the maximum cell promotion was observed on TK-10 (renal cancer), which showed 15.84% growth promotion (84.16% growth inhibition) at one-dose assay. The compound **10** was found to be highly active on SK-MEL-5 (melanoma) with cell promotion of −86.52% followed by UACC-62 (melanoma) with cell promotion of −84.39% and COLO 205 (colon cancer) with cell promotion of −84.05% while the maximum cell promotion was observed on UACC-257 (melanoma), which showed 16.03% growth promotion (83.97% growth inhibition) at one-dose assay. Compound **11** was found to be highly active on CAKI-1 (renal cancer) with cell promotion of −34.77% followed by MDA-MB-435 (melanoma) with cell promotion of −30.53% and SF-295 (CNS cancer) with cell promotion of −29.68% while the maximum cell promotion was observed on UACC-257 (melanoma), which showed 87.13% growth promotion (12.87% growth inhibition) at one-dose assay. Compound **12** was found to be highly active on HT29 (colon cancer) with cell promotion of −6.41% followed by MDA-MB-468 (breast cancer) with cell promotion of −6.34% and CCRF-CEM (leukemia) with cell promotion of −16.72% while the maximum cell promotion was observed on TK-10 (renal cancer), which showed 102.74% growth promotion at one-dose assay. Compound **13** was found to be highly active on HT29 (colon cancer) with cell promotion of −76.24% followed by 786-0 and A498 (renal cancer) with cell promotion of −58.93% and −56.92%, respectively, while the maximum cell promotion was observed on NCI/ADR-RES (ovarian cancer), which showed 39.32% growth promotion (12.87% growth inhibition) at one-dose assay. Among the 3,5-bis(4-hydroxy-3-methylstyryl)-*N*-(substituted phenyl)-1*H*-pyrazole-1-carboxamide analogues (**1–9**), Compound **8** was found to be the most active compound of the series with mean growth percent of −19.19 followed by compound **5** which showed mean growth percent of −12.25 and compound **1**, which showed mean growth percent of −4.66. Among the 3,5-bis(4-hydroxy-3-methylstyryl)-1*H*-pyrazole-1-yl (substituted phenyl)methanone analogues (**10-11**), Compound **10** showed maximum activity with mean growth percent of −28.71 while compound **13** was found to be the most active compound among dihydropyrimidine analogues (**12–14**) which showed mean growth percent of −5.46. Overall, the most active compound of the series was compound **10**, and most of the 3,5-bis(4-hydroxy-3-methylstyryl)-*N*-(substituted phenyl)-1*H*-pyrazole-1-carboxamide analogues possessed potent anticancer activity. Based on our finding, we can conclude that electronegative group at position 2 (i.e., 2-chloro) on *N*-substituted phenyl ring among 3,5-bis(4-hydroxy-3-methylstyryl)-*N*-(substituted phenyl)-1*H*-pyrazole-1-carboxamide analogues (**1–9**) showed more anticancer activity than at position 4 (i.e., 4-chloro and 4-fluoro). Electron releasing group, such as 2,4-dimethyl and 4-methyl on *N*-substituted phenyl ring, increased anticancer activity, and the activity was found to be more if the number of electron releasing groups was increased. Among the 3,5-bis(4-hydroxy-3-methylstyryl)-1*H*-pyrazole-1-yl (substituted phenyl)methanone analogues (**10** and **11**), the activity was found to be more when there was no substitution in the phenyl ring. Among six member dihydropyrimidine analogues (**12–14**), compound with X substitution as “NH” showed more activity than as “O” substitution.

**Figure 2:**
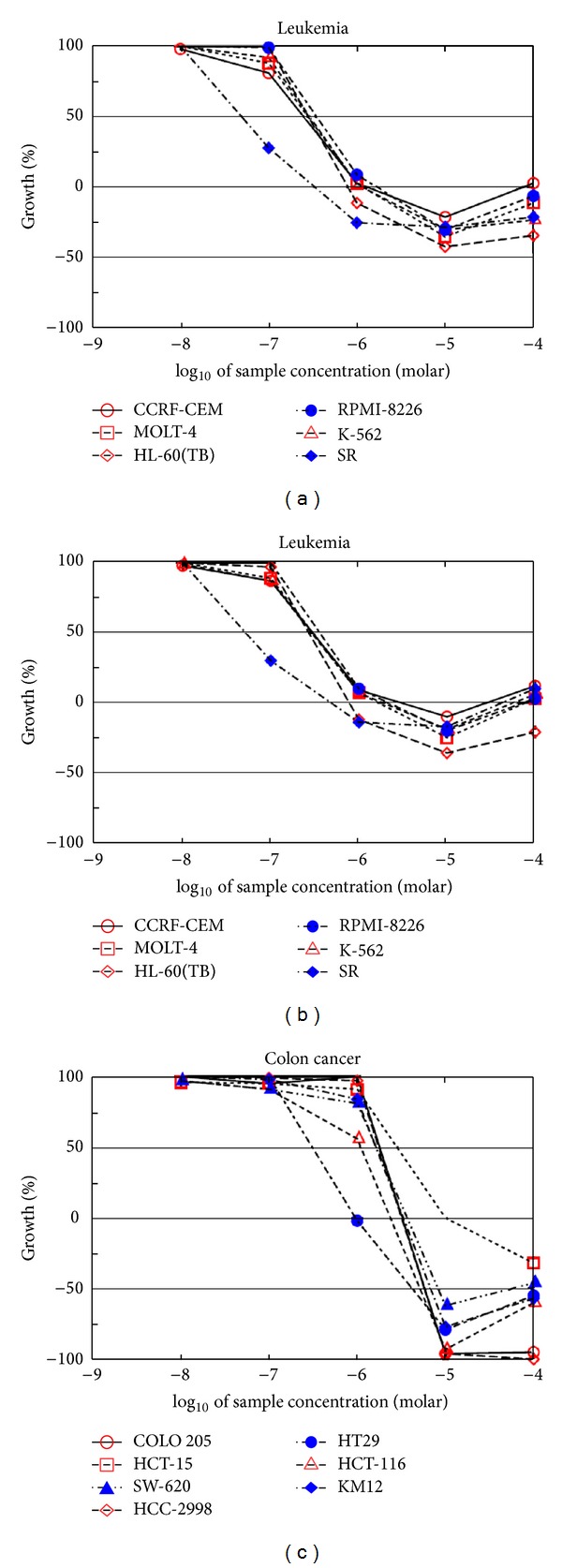
(a) Five-dose anticancer screening of compound **8** on leukemia cell line. (b) Five-dose anticancer screening of compound **10** on leukemia cell line. (c) Five-dose anticancer screening of compound **13** on colon cancer cell line.

The results of 5-dose assay of the most active compounds among their respective series are given in Table 3. Compound **8** presented GI_50_ ranging between 0.004 and 2.04 *μ*M. The best results were recorded on the leukemia cell lines with values ranging from 0.004 to 0.364 *μ*M (Figure 2(a)). A number of 3 tested cancer cell lines presented TGI value with >100 *μ*M, the best result value being noted on the MDA-MB-435 (melanoma) with value 0.383 *μ*M. Only in 23 cell lines, compound **8** registered LC_50_ value with >100 *μ*M. Compound **10** presented GI_50_ ranging between 0.0079 and 1.86 *μ*M. The best results were recorded on the leukemia cell line with value ranging from 0.0353 to 0.347 *μ*M (Figure 2(b)). Four of the tested cancer cell lines presented TGI value with >100 *μ*M, the best result value being noted on the MDA-MB-435 (melanoma) with value 0.00815 *μ*M. Only in 27 cell lines, compound **10** registered LC_50_ value with >100 *μ*M. Compound **13** presented GI_50_ ranging between 0.524 and 3.39 *μ*M. The best results were recorded on the colon cancer cell line with values ranging from 0.543 to 2.18 *μ*M (Figure 2(c)). Only one tested cancer cell line presented TGI value with >100 *μ*M, the best result value being noted on the HT29 (colon cancer) with value 0.948. In 28 cell lines, compound **13** registered LC_50_ value with >100 *μ*M.

Some of the synthesized compounds were evaluated for EGFR tyrosine kinase inhibitory activity by recombinant tyrosine kinase assay using an ELISA-based assay with poly(Glu, Tyr, 4 : 1) as a substrate [37]. The results showed compounds **8**, **10**, and **13**, shows inhibition of EGFR tyrosine kinase with IC_50_ values of 35.5, 29.2, and 55.7 *μ*M respectively.

### 3.3. Molecular Docking

**Figure 3:**
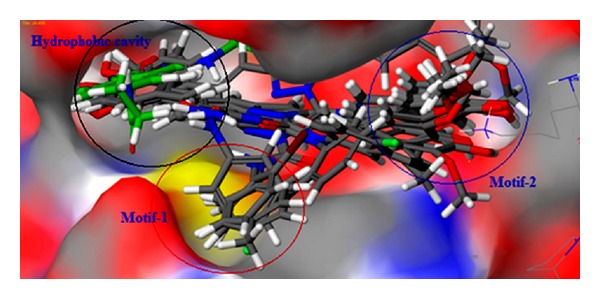
Green colour stick model indicates the reference ligand and atom macromodel stick indicates our ligands.

The binding site of the reference inhibitor is well defined by hydrophobic cavity. The EGFR tyrosine kinase binding site contains the important residues Cys797 and Thr790, re docking of reference ligand shown that above residues are important for hydrophobic interactions with the same [38]. The hydrophobic cavity with the residues Leu788, Met766, Lys745, Glu762, Thr854, and Met793 makes the binding site attractive for the design of new inhibitors. Based on our knowledge, we designed some class of new pyrazole (**1–11**), dihydropyrimidine analogues (**12–14**) for EGFR tyrosine kinase inhibition. But the binding sites of our new molecules were found to be a little different with comparison to reference inhibitor which is reported in the crystal structure of EGFR tyrosine kinase (PDB: 2J5F). These ligands' interactions were shown in three different motifs. The first one is hydrophobic cavity in which the substituted methoxy, hydroxy phenyl ring of the ligand is showing good interactions as reference inhibitor. The next binding motif contains the target residue Cys797, the *N*-substituted pyrazole derivatives, and six membered (dihydropyrimidine) are binding at this site. Most of these interactions were hydrophobic. The last binding motif contains the residues Lys875 and Asp831 which lie in side chain of the protein and showed the side chain hydrogen bonding with the designed molecules. The docking score and Emodel score of some of the compounds selected for anticancer activity by NCI are given in Table 4, and binding modes are shown in Figure 3.

**Figure 4:**
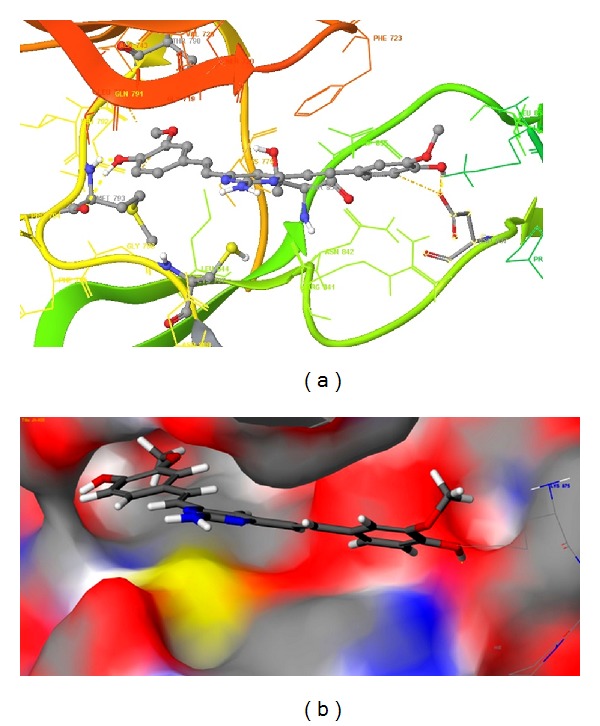
(a) Compound **13** is represented with macromodel stick model. The hydrogen bonds are shown with yellow colour dotted lines and hydrophobic interactions with orange dotted lines. (b) Compound **13** at binding site on surface.

**Figure 5:**
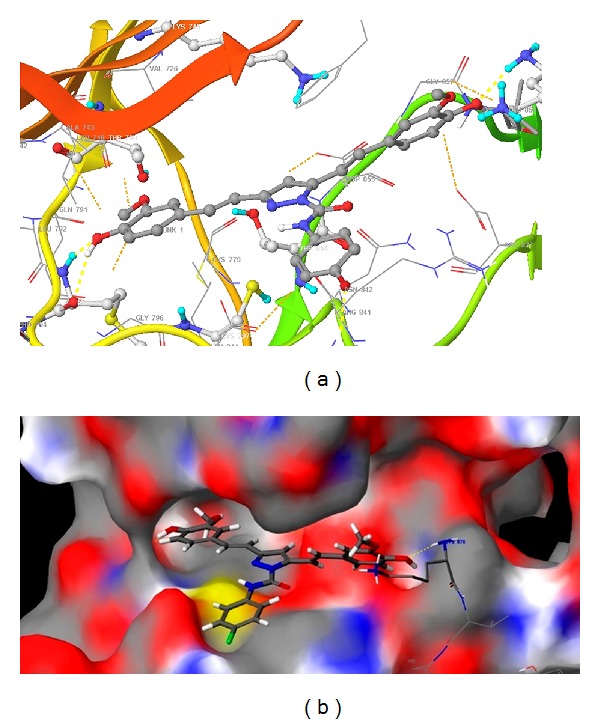
(a) Compound **3** is represented with macromodel stick model. The hydrophobic cavity residues are represented with magenta stick model. The hydrogen bonds are shown with yellow colour dotted lines and hydrophobic interactions with orange dotted lines. (b) Compound **3** at binding site on surface.

Compound **13** showed the best hydrophobic interaction with various residues such as Cys797 Met 793, Leu 844, Leu 792, Ala 743, and Val 726 (Figures 4(a) and 4(b)). Negatively charged Asp 837 interacted with methoxy, hydroxy phenyl ring (4-hydroxy-3-methoxyphenyl). Arg 841 showed *π*-*π* cationic interaction. The Met 793 present in the hydrophobic cavity showed H-bond backbone interaction and H-bond (side chain) interaction. The positively charged Lys 845 and Arg 841 destabilized interactions. The interactions of compound **13** with the EGFR kinase receptor shown in ribbon diagram, the hydrophobic cavity, is indicated with yellow colour loop, motif-1 is indicated with green colour beta sheet, and the motif-2 is indicated with green colour loop. The other ligands were also having good interactions with the hydrophobic cavity and binding motifs the following residues leu718, Pro794, Met793, Ala743, Leu844, Val726, and Cys797 were present around the ligand. Thr790, Gln791, having polar characteristics and Met 793 were responsible for backbone H-bond interaction as well as H-bond (side chain) interaction. Asp855 and Asp837 showed charged interactions like destabilizing contacts. Positively charged Lys875 and Arg841 were found to have *π*-*π* cationic interaction and were present in binding motif, making different binding patterns of reported ligands. The docking study for compound **3** is given in Figures 5(a) and 5(b).

## 4. Conclusion

The novel series of curcumin analogues (pyrazoles) were synthesized in satisfactory yields. The anticancer activity showed promising results. The studies confirmed compound **10** as potent lead compound for drug discovery and further optimization. The curcumin analogues discovered in this study may provide valuable therapeutic intervention for the treatment of cancer disease. The pyrazole discovered in this study may provide valuable therapeutic intervention for the treatment of cancer.

## Supplementary Material

All the structures were drawn by using Maestro. Their structures were minimized using Macromodel minimization panel using the OPLS-2005 force field and GB/SA water model with a constant dielectric of 1.0 Polak-Ribiere frst derivative, conjugate gradient minimization was employed with maximum iterations of 1000 and convergence threshold of a gradient to < 0.05 kJ/Å-mol. LigPrep2.0 module of Schrodinger was used to generate possible ionization states at target pH 7.0±2.0. All possible tautomeric states at this pH were also generated using the tautomerizer module of LigPrep2.0. The resulting structures were saved in ∗.mae format for further experiments.

## Figures and Tables

**Table 1:** Physical constants of the curcumin analogues (**1–14**).

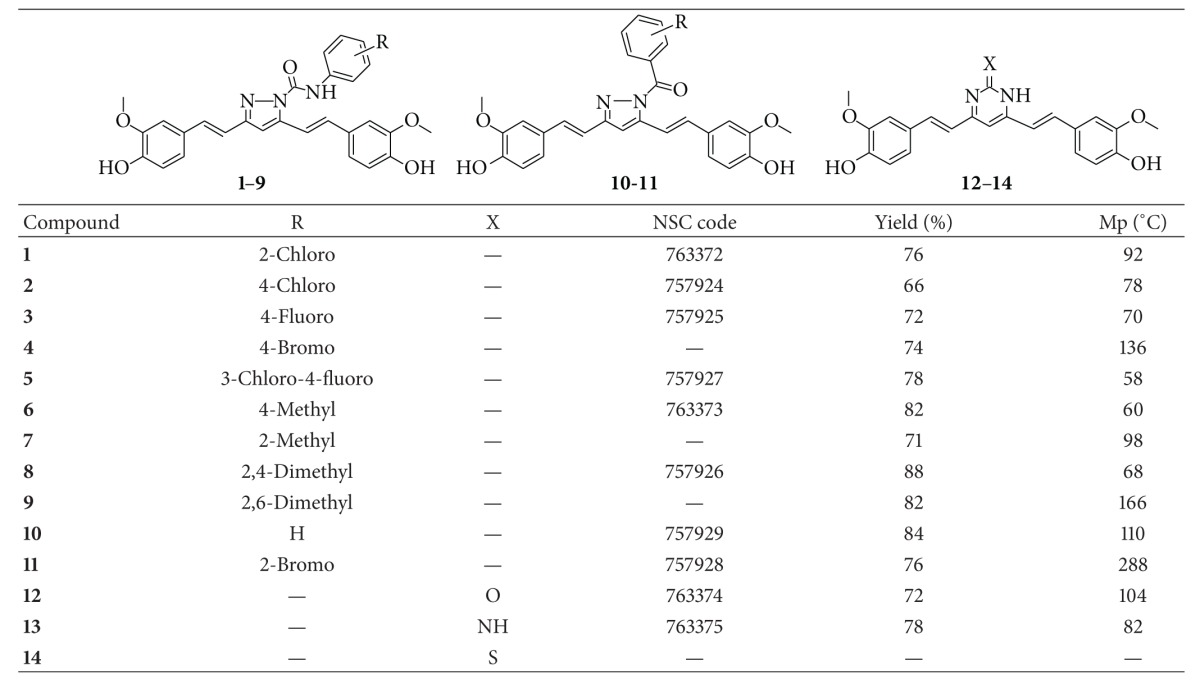

**Table 2:** Sixty human tumor cell lines anticancer screening data of curcumin analogues (**1–14**).

Panel/cell line	Growth percent in one-dose assay									
	**1**	**2**	**3**	**5**	**6**	**8**	**10**	**11**	**12**	**13**
Leukemia										
CCRF-CEM	−4.54	4.45	6.82	0.87	0.44	1.54	−5.12	3.52	16.72	1.68
HL-60(TB)	−24.28	−20.94	−21.36	−28.92	−28.17	−30.36	−42.20	−23.89	61.33	3.67
K-562	2.27	−7.30	0.22	−3.90	2.29	−7.18	−15.52	3.62	42.03	5.49
MOLT-4	−4.57	−2.00	−3.56	−17.67	0.11	−20.68	−39.87	−6.53	36.34	4.17
RPMI-8226	−22.31	2.91	3.32	−11.32	−14.46	−16.73	−33.60	14.25	62.49	2.88
SR	−17.72	1.52	2.53	1.44	−14.65	−2.07	−13.89	−4.77	27.24	−7.94
Non-small-cell lung cancer										
A549/ATCC	8.75	4.03	12.02	5.90	4.71	−3.98	8.40	41.73	71.68	10.09
EKVX	17.68	16.85	26.87	−17.67	20.29	−14.72	−38.79	30.55	71.60	22.16
HOP-62	8.39	17.06	30.22	−7.78	11.62	−35.66	−67.73	43.53	70.77	4.85
HOP-92	−14.42	−4.70	−3.45	NT	−15.38	−27.02	NT	NT	33.70	−21.24
NCI-H226	−3.72	5.73	15.88	−4.32	−0.89	−3.70	−8.19	38.48	76.20	11.70
NCI-H23	−25.82	3.16	10.74	−12.87	−10.41	−26.09	NT	17.66	62.85	−2.79
NCI-H322M	NT	22.13	27.32	12.63	NT	8.13	−3.64	39.48	NT	NT
NCI-H460	7.39	9.41	8.35	4.43	10.14	1.87	−0.07	9.15	69.70	5.54
NCI-H522	−18.66	−4.94	−6.04	10.89	−19.34	6.31	−30.23	4.20	54.59	−41.31
Colon cancer										
COLO 205	−73.49	7.57	8.73	59.43	−25.81	−65.55	−84.05	23.87	51.81	−50.38
HCC-2998	2.83	4.55	6.92	9.75	2.19	4.28	5.82	40.94	70.99	0.93
HCT-116	1.16	4.61	4.95	2.15	1.60	1.48	−5.83	6.90	34.75	0.33
HCT-15	15.05	7.86	10.63	6.64	15.22	3.84	−3.38	16.21	81.79	29.55
HT29	−34.95	−11.07	−41.84	−33.57	−22.35	−38.54	−52.39	0.38	−6.41	−76.24
KM12	0.09	3.53	2.44	0.08	−1.13	−9.35	−27.96	4.98	29.26	−29.25
SW-620	6.99	9.56	10.52	2.03	8.12	−10.94	−22.72	20.86	43.58	1.86
CNS cancer										
SF-268	−6.38	13.87	20.12	6.57	−11.50	3.80	−12.52	27.97	48.19	3.56
SF-295	2.83	−21.89	−21.61	−55.32	0.53	−51.02	−71.47	−29.68	92.76	−5.25
SF-539	4.06	0.16	3.68	−16.58	5.95	−19.58	−39.79	4.02	69.02	11.95
SNB-19	15.49	9.25	20.15	4.42	20.27	2.45	1.18	36.22	82.60	23.19
SNB-75	−4.71	18.75	26.93	−12.60	9.53	−30.53	−53.18	27.28	44.32	5.50
U251	2.33	4.31	13.38	−1.59	6.19	−12.27	−1.47	35.06	55.64	2.97
Melanoma										
LOX IMVI	1.22	3.04	4.00	−20.87	1.89	−29.79	−37.11	9.29	49.04	−16.09
MALME-3M	8.27	3.94	9.15	−16.80	17.27	−21.70	−38.98	23.43	53.57	−28.85
M14	0.26	4.04	2.69	−21.86	2.30	−38.14	−55.95	5.42	75.46	−26.13
MDA-MB-435	4.08	2.46	−2.32	−25.94	−1.64	−40.91	−67.34	−30.53	56.89	−8.12
SK-MEL-2	−12.27	−23.52	−27.07	−25.00	−13.98	−27.22	−48.11	−0.33	81.15	−21.01
SK-MEL-28	12.10	12.72	16.70	7.87	13.22	5.57	−6.73	28.64	50.89	9.55
SK-MEL-5	−23.76	−29.66	−8.36	−73.86	1.16	−83.44	−86.52	−1.91	70.74	−18.40
UACC-257	1.28	3.11	13.66	−15.89	4.80	−21.42	16.03	87.13	62.46	−1.90
UACC-62	1.04	4.06	2.01	−37.42	0.49	−48.47	−84.39	3.48	63.31	−57.66
Ovarian cancer										
IGROV1	−6.58	9.93	17.41	4.57	4.76	−1.75	−21.18	24.95	93.12	3.42
OVCAR-3	−26.77	−10.97	3.89	−26.44	−34.15	−37.33	−46.92	12.55	59.34	−7.57
OVCAR-4	6.59	13.25	35.55	9.26	16.08	7.87	4.49	49.80	53.98	18.31
OVCAR-5	13.18	10.94	31.32	0.88	20.53	−4.24	−7.74	84.49	101.67	5.83
OVCAR-8	1.50	−19.83	0.47	−44.00	−2.98	−34.66	−13.11	27.73	58.71	9.33
NCI/ADR-RES	20.83	17.46	27.91	12.01	20.50	7.64	3.18	28.13	90.66	39.32
SK-OV-3	−33.32	−0.37	−18.66	−31.16	−29.09	−63.58	−53.08	16.55	71.63	−17.39
Renal cancer										
786-0	−14.80	−2.20	−6.90	−32.92	−1.18	−59.07	−76.67	9.91	82.57	−58.93
A498	−11.83	−0.60	31.49	−13.65	12.02	−14.70	−25.17	56.41	62.76	−56.92
ACHN	2.83	9.29	20.22	5.73	16.12	5.25	2.77	25.12	89.36	13.46
CAKI-1	7.29	0.13	−7.81	−39.10	8.65	−36.63	−60.45	−34.77	88.61	19.24
RXF 393	−50.32	−53.60	−43.84	−57.61	−47.46	−67.29	−70.03	−3.83	23.75	−63.92
SN 12C	14.20	9.00	17.95	2.06	18.30	0.47	−3.11	14.23	62.26	22.14
TK-10	16.15	23.10	38.12	24.04	28.55	15.86	6.18	65.02	102.74	21.38
UO-31	−34.84	2.71	14.05	−16.95	0.97	−38.74	−44.64	23.86	54.25	3.16
Prostate cancer										
PC-3	6.54	4.24	9.01	−14.26	8.08	−10.82	−33.14	12.92	61.01	12.99
DU-145	1.23	11.66	6.77	−11.84	2.20	−26.64	−45.74	28.87	80.37	13.04
Breast cancer										
MCF7	4.41	5.36	9.49	2.06	6.30	−3.78	−5.71	20.22	52.10	7.59
MDA-MB-231/ATCC	17.71	14.35	33.61	12.24	19.94	10.25	5.23	39.69	54.19	3.39
HS 578T	−4.71	8.23	20.04	−12.92	−15.97	−14.33	−12.98	3.59	53.91	−1.00
BT-549	−26.40	−14.35	−8.33	−24.17	−23.00	−33.81	−47.16	14.49	55.03	−24.41
T-47D	4.55	18.50	31.15	2.29	5.65	−22.55	−23.93	48.99	59.14	1.57
MDA-MB-468	−24.19	−26.40	−14.60	−27.56	−29.14	−30.34	−31.86	2.97	6.34	−35.04

Mean	−**4.66**	**1.81**	**7.23**	−**12.25**	−**0.66**	−**19.19**	−**28.71**	**19.03**	**59.94**	−**5.46**

Range	**94.32**	**76.70**	**81.96**	**97.90**	**76.01**	**99.30**	**102.55**	**121.90**	**109.15**	**115.56**

NT: not tested.

**Table 3:** NCI *in vitro* testing results of compounds of the compounds **8**, **10**, and **13** at five-dose level in *µ*M.

S. no.	Panel/cell line	**8 (NSC 757926)**			**10 (NSC 757929)**			**13 (NSC 763375)**		
		GI_50_	TGI	LC_50_	GI_50_	TGI	LC_50_	GI_50_	TGI	LC_50_
(1)	Leukemia									
	CCRF-CEM	0.232	>100	>100	0.127	>100	>100	0.543	NT	>100
	HL-60(TB)	0.237	0.806	>100	0.0884	NT	>100	2.18	6.17	>100
	MOLT-4	0.364	NT	>100	0.347	>100	>100	1.01	5.19	>100
	RPMI-8226	0.336	NT	>100	0.275	NT	>100	1.29	4.18	>100
	SR	0.004	>100	>100	0.0353	>100	>100	0.72	3.58	>100
(2)	Non-small cell lung cancer									
	A549/ATCC	0.445	3.63	56.3	0.403	1.91	>100	2.22	6.04	76.4
	EKVX	0.771	5.51	>100	0.702	2.38	NT	2.22	6.83	>100
	HOP-62	0.497	1.63	4.21	0.441	1.62	4.23	1.96	4.28	NT
	HOP-92	0.538	3.39	>100	0.35	3.32	>100	1.26	5.19	>100
	NCI-H226	0.389	2.1	12.9	0.382	1.83	7.68	1.83	4.34	12
	NCI-H23	0.275	1.25	>100	0.113	1.61	>100	1.8	4.24	NT
	NCI-H322M	0.571	2.8	85.8	0.615	2.49	NT	2.22	5.39	>100
	NCI-H460	0.306	NT	>100	0.274	NT	>100	2.05	5.20	>100
	NCI-H522	0.13	0.616	NT	0.286	7.59	NT	1.24	2.79	6.31
(3)	Colon cancer									
	COLO 205	2.05	3.62	6.38	1.86	3.34	5.97	1.8	3.24	5.85
	HCC-2998	0.64	5.8	>100	0.591	2.71	>100	1.75	3.19	5.79
	HCT-116	0.323	NT	NT	0.269	1.14	NT	1.1	2.39	5.18
	HCT-15	0.335	1.66	NT	0.167	1.52	NT	2.82	10.3	>100
	HT29	0.696	2.8	9.94	0.463	3.19	>100	3.14	0.948	4.21
	KM12	0.298	1.25	NT	0.0092	1.19	NT	1.63	3.32	6.79
	SW-620	0.441	NT	>100	0.0079	NT	>100	1.65	3.68	NT
(4)	CNS cancer									
	SF-268	0.571	NT	NT	0.256	NT	NT	2.11	15.6	>100
	SF-295	0.546	NT	>100	0.538	NT	NT	1.93	4.54	>100
	SF-539	0.354	1.5	NT	0.0604	1.53	NT	2.05	5.73	>100
	SNB-19	0.634	2.01	NT	0.572	2.12	NT	1.81	7.95	>100
	SNB-75	0.416	1.82	NT	0.0099	1.87	NT	1.26	3.34	8.84
	U251	0.471	1.59	NT	0.428	1.70	NT	1.79	5.69	6.63
(5)	Melanoma									
	LOX IMVI	0.284	1.49	NT	0.0626	1.56	NT	1.27	2.59	NT
	MALME-3M	0.563	1.98	NT	0.525	2.17	NT	2.71	6.76	>100
	M14	0.372	1.41	NT	0.155	1.48	NT	1.65	3.44	NT
	MDA-MB-435	0.0448	0.383	NT	0.0175	0.00815	>100	1.71	NT	>100
	SK-MEL-2	0.366	1.82	NT	0.266	2.03	>100	1.98	4.1	NT
	SK-MEL-28	0.509	2.47	8.61	0.176	2.72	9.05	1.85	4.53	26
	SK-MEL-5	0.317	1.29	3.67	0.171	1.30	3.69	1.49	2.89	5.59
	UACC-257	1.54	5.47	31.9	1.14	4.29	53.3	1.53	3.5	8
	UACC-62	0.413	1.65	4.89	0.371	1.64	NT	1.49	3.23	NT
(6)	Ovarian cancer									
	IGROV1	0.454	2.62	>100	0.483	NT	>100	2.95	7.08	>100
	OVCAR-3	0.412	1.72	>100	0.242	1.16	>100	2.65	8.62	63.5
	OVCAR-4	0.481	3.32	>100	0.530	6.76	>100	1.71	11.5	71.1
	OVCAR-5	1.51	6.85	>100	1.27	4.51	>100	2.03	4.33	9.22
	OVCAR-8	0.563	2.35	9.71	0.479	3.78	>100	2.19	7.24	>100
	NCI/ADR-RES	0.338	4.01	>100	0.0874	3.85	>100	2.88	18.8	>100
	SK-OV-3	0.419	1.6	7.28	0.358	1.35	3.88	2.6	8.23	>100
(7)	Renal cancer									
	786-0	0.549	1.76	4.61	0.688	2.03	4.99	1.7	4	NT
	A498	0.575	1.98	4.63	0.697	2	4.65	1.51	3.26	7.04
	ACHN	0.412	2.11	>100	0.131	1.69	NT	2.54	7.88	>100
	CAKI-1	0.341	5.49	>100	0.282	3.24	>100	2.23	6.46	>100
	RXF 393	0.227	0.653	2.72	0.263	0.878	3.60	1.5	3.2	NT
	SN 12C	0.339	1.88	NT	0.313	2.20	>100	2.74	>100	>100
	TK-10	0.424	2	7.15	0.446	1.76	5.19	3.39	9.54	5.34
	UO-31	0.362	1.74	5.93	0.258	1.65	4.73	2.09	7.57	>100
(8)	Prostate cancer									
	PC-3	0.349	5.78	>100	0.331	12.7	>100	1.48	5.57	>100
	DU-145	0.419	1.44	NT	0.389	1.51	NT	2.4	6.31	88
(9)	Breast cancer									
	MCF7	0.317	1.37	4.86	0.162	1.6	6.57	1.81	6.33	38.8
	MDA-MB-231/ATCC	0.473	4.71	>100	0.404	4.83	>100	1.45	4.08	>100
	HS 578T	0.295	>100	>100	0.0779	>100	>100	0.524	2.99	>100
	BT-549	0.693	16.8	>100	0.631	22.8	>100	1.3	3.52	NT
	T-47D	0.79	2.94	NT	0.793	2.93	9.79	2.83	9.55	>100
	MDA-MB-468	0.248	0.825	63.1	0.276	0.969	>100	NT	NT	NT

NT: not tested.

**Table 4:** The docking score and E model score of the reported compounds.

S. no.	Ligand	Glide score	E model score
1	Reference	−8.288	−68.491
2	**1**	−7.538	−80.638
3	**2**	−5.935	−83.181
4	**3**	−6.903	−76.437
5	**5**	−7.272	−80.080
6	**6**	−6.721	−81.342
7	**8**	−6.725	−71.692
8	**10**	−6.647	−76.710
9	**11**	−6.766	−78.595
10	**12**	−7.677	−56.626
11	**13**	−7.702	−47.220
