# Supplementary material for: Synthesis, Characterisation, and In Vitro Anticancer Activity of Curcumin Analogues Bearing Pyrazole/Pyrimidine Ring Targeting EGFR Tyrosine Kinase
Source: Biomed Res Int. 2013 Sep 9;2013:239354. doi: 10.1155/2013/239354 (PMC3781846; doi:10.1155/2013/239354)

Synthesis, characterisation and *in vitro* anticancer activity of curcumin analogues bearing pyrazole/pyrimidine ring targeting EGFR tyrosine kinase

Mohamed Jawed Ahsan[1,*], *Habibullah Khalilullah*[2], *Sabina Yasmin*[3], *Surender Singh Jadav*[3], *Jeyabalan Govindasamy*[2]

[1]Department of Pharmaceutical Chemistry, Maharishi Arvind College of Pharmacy, Ambabari, Jaipur, Rajasthan 302 023, India

[2]Department of Pharmaceutical Chemistry, Alwar Pharmacy College, Alwar, Rajasthan 301 030, India

[3]Department of Pharmaceutical Chemistry, Birla Institute of Technology, Mesra, Ranchi, Jharkhand 835 215, India

Supplementary Materials

A series of 14, novel curcumin analogues has been synthesized. There were 10 tested compounds among 14 synthesized compounds, which showed potent anticancer activity in both one dose and 5-dose assays. The five dose assay for the compounds **8**, **10**, and **13** is described and given in the article in μM while for rest of the compounds (**1**, **2**, **3**, **5**, **6**, **11**, and **12**) the results of five dose assay can be found online as supplementary materials.

National Cancer Institute Developmental Therapeutics Program<br>Mean Graphs	NSC :763372/1	Units :Molar	SSPL :0Y1V	EXP. ID :1202NS13
	Report Date :April 25, 2012		Test Date :February 21, 2012	

Panel/Cell Line	Log_{10}GI50	Log_{10}TGI	Log_{10}LC50
**Leukemia**			
CCRF-CEM	-6.67	-6.07	> -4.00
HL-60(TB)	-6.50	-6.05	> -4.00
K-562	-6.58	-5.75	> -4.00
MOLT-4	-6.48	-5.86	> -4.00
RPMI-8226	-6.63	-6.17	
SR	-7.57	-7.07	> -4.00
**Non-Small Cell Lung Cancer**			
A549/ATCC	-6.49	-5.68	-4.72
EKVX	-6.17	-5.42	-4.43
HOP-62	-6.27	-5.73	-5.34
HOP-92	-6.50	-5.79	-4.76
NCI-H226	-6.21	-5.56	-4.48
NCI-H23	-6.53	-5.72	> -4.00
NCI-H322M	-6.21	-5.20	-4.19
NCI-H460	-6.48	-5.86	-4.68
NCI-H522	-7.21	-6.39	-5.58
**Colon Cancer**			
COLO 205	-5.75	-5.50	-5.25
HCC-2998	-6.19	-5.23	-4.57
HCT-116	-6.51	-5.98	
HCT-15	-6.44	-4.98	-4.21
HT29	-6.54	-6.15	-5.47
KM12	-6.53	-5.82	-5.21
SW-620	-6.44	-5.73	-4.96
**CNS Cancer**			
SF-268	-6.25	-5.32	> -4.00
SF-295	-6.16	-5.35	-4.51
SF-539	-6.34	-5.65	-5.11
SNB-19	-6.04	-5.23	-4.54
SNB-75	-6.61	-6.01	-5.30
U251	-6.38	-5.77	-5.28
**Melanoma**			
LOX IMVI	-6.71	-5.86	-5.20
MALME-3M	-6.36	-5.58	
M14	-6.61	-5.89	-5.32
MDA-MB-435	-7.56	-7.00	-4.97
SK-MEL-28	-6.30	-5.39	-4.65
SK-MEL-5	-6.56	-5.89	-5.44
UACC-257	-5.96	-5.41	-4.76
UACC-62	-6.33	-5.43	> -4.00
**Ovarian Cancer**			
IGROV1	-6.30	-5.57	> -4.00
OVCAR-3	-6.33	-5.43	-4.49
OVCAR-5	-5.48	-5.03	> -4.00
OVCAR-8	-6.40	-5.77	-5.02
NCI/ADR-RES	-6.61	-5.10	> -4.00
SK-OV-3	-6.41	-5.66	-4.66
**Renal Cancer**			
786-0	-6.34	-5.76	-5.29
A498	-6.31	-5.68	-5.22
ACHN	-6.43	-4.91	-4.09
CAKI-1	-6.70	-6.12	> -4.00
RXF 393	-6.48	-6.09	-5.42
SN12C	-6.41	-4.80	> -4.00
TK-10	-6.29	-5.56	-4.72
UO-31	-6.64	-5.91	
**Prostate Cancer**			
PC-3	-6.50	-5.75	-4.91
DU-145	-6.27	-5.74	-5.30
**Breast Cancer**			
MCF7	-6.54	-5.85	-5.32
MDA-MB-231/ATCC	-6.12	-4.87	> -4.00
HS 578T	-7.38	-6.51	-4.09
BT-549	-6.52	-5.42	> -4.00
T-47D	-6.00	-5.58	-5.16
_MID	-6.45	-5.69	-4.65
Delta	1.12	1.38	0.93
Range	2.09	2.27	1.58

GI50

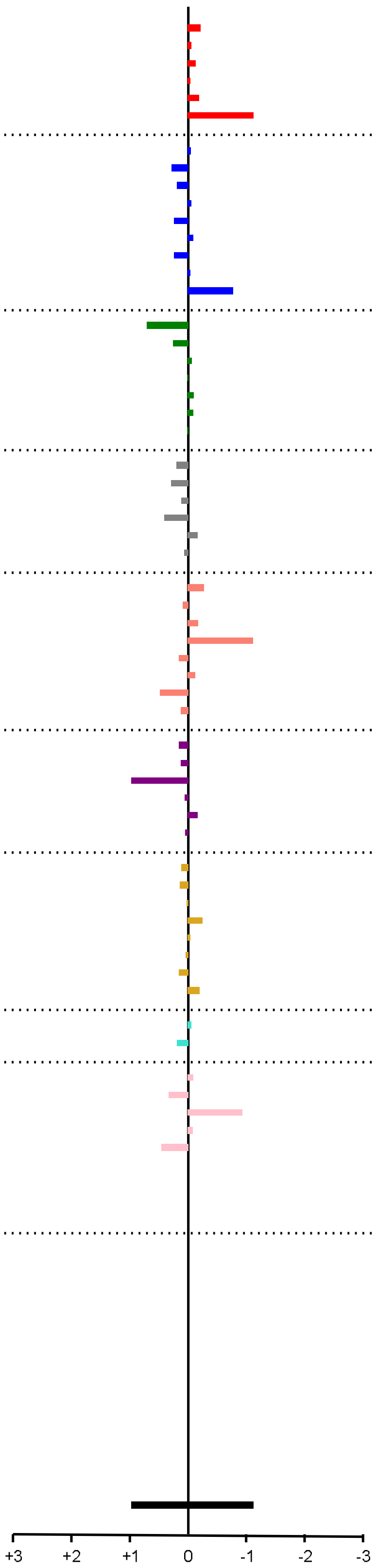

TGI

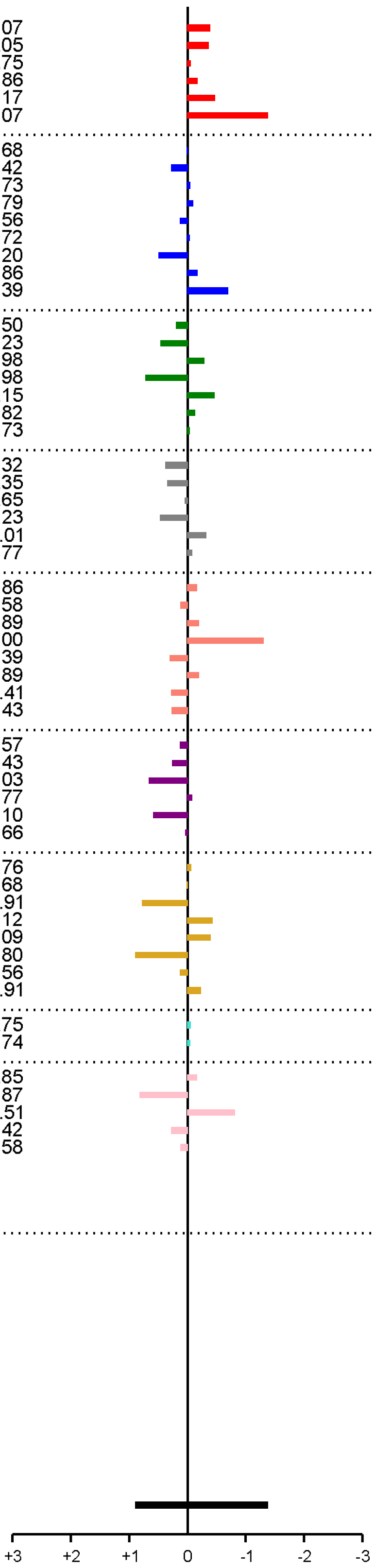

LC50

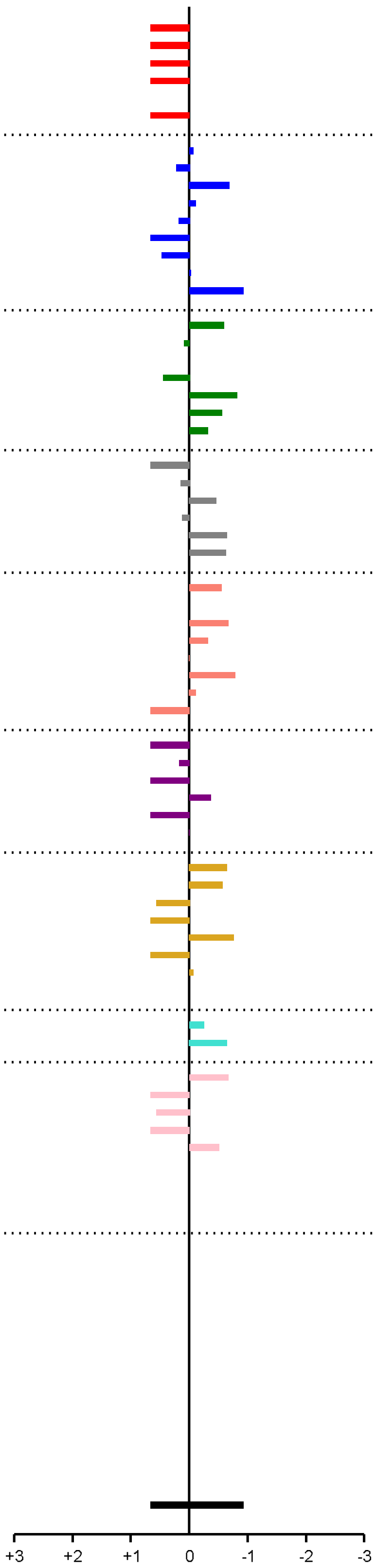

National Cancer Institute Developmental Therapeutics Program Mean Graphs	NSC :757924/1	Units :Molar	SSPL :0Y1V	EXP. ID :1108RS99
	Report Date :September 27, 2011		Test Date :August 08, 2011	

Panel/Cell Line	Log_{10}GI50	Log_{10}TGI	Log_{10}LC50
**Leukemia**			
CCRF-CEM	-6.52	> -4.00	> -4.00
HL-60(TB)	-6.54	-5.98	> -4.00
MOLT-4	-6.38	-5.24	> -4.00
RPMI-8226	-6.46	-5.56	> -4.00
SR	-7.21	> -4.00	> -4.00
**Non-Small Cell Lung Cancer**			
A549/ATCC	-6.15	-4.97	-4.39
EKVX	-5.82	-4.93	-4.12
HOP-62	-6.27	-5.72	-5.26
HOP-92	-5.66	-5.14	> -4.00
NCI-H226	-5.73	-5.15	-4.52
NCI-H23	-6.47	-5.86	
NCI-H322M	-6.11	-5.03	-4.31
NCI-H460	-6.50	-5.67	> -4.00
NCI-H522	-6.67	-6.10	-4.29
**Colon Cancer**			
COLO 205	-5.69	-5.45	-5.21
HCC-2998	-5.82	-5.12	-4.49
HCT-116	-6.44	-5.73	-4.44
HCT-15	-6.34	-4.93	> -4.00
HT29	-5.60	-5.20	> -4.00
KM12	-6.47	-5.84	-4.92
SW-620	-6.35	-5.60	> -4.00
**CNS Cancer**			
SF-268	-6.29	-5.44	-4.54
SF-295	-5.80	-4.94	> -4.00
SF-539	-6.32	-5.65	-5.08
SNB-19	-6.19	-4.98	-4.35
SNB-75	-6.38	-5.59	> -4.00
U251	-6.26	-5.26	-4.34
**Melanoma**			
LOX IMVI	-6.36	-5.62	> -4.00
MALME-3M	-6.22	-5.50	-4.70
M14	-6.42	-5.82	-4.75
MDA-MB-435	-6.87	-6.43	-5.97
SK-MEL-2	-6.42	-5.71	> -4.00
SK-MEL-28	-5.94	-5.34	-4.59
SK-MEL-5	-6.46	-5.87	-5.39
UACC-257	-5.85	-5.19	-4.53
UACC-62	-6.24	-5.62	-5.11
**Ovarian Cancer**			
IGROV1	-6.23	-5.31	> -4.00
OVCAR-3	-6.45	-5.98	-4.23
OVCAR-4	-6.01	-5.15	> -4.00
OVCAR-5	-5.61	-5.12	> -4.00
OVCAR-8	-6.13	-5.42	-4.15
NCI/ADR-RES	-6.44	-5.45	> -4.00
SK-OV-3	-6.35	-5.77	-5.19
**Renal Cancer**			
786-0	-6.28	-5.75	-5.32
A498	-5.73	-5.28	-4.72
ACHN	-6.28	-4.94	-4.32
CAKI-1	-6.43	-4.10	> -4.00
RXF 393	-6.60	-6.12	-5.50
SN12C	-6.34	-4.67	> -4.00
TK-10	-6.18	-5.21	-4.54
UO-31	-6.41	-5.37	-4.39
**Prostate Cancer**			
PC-3	-6.38	-5.17	> -4.00
DU-145	-6.42	-5.83	-5.16
**Breast Cancer**			
MCF7	-6.36	-5.48	-4.40
MDA-MB-231/ATCC	-6.20	-5.17	> -4.00
HS 578T	-6.36		> -4.00
BT-549	-6.12	-5.34	> -4.00
T-47D	-5.91	-5.30	-4.35
MDA-MB-468	-6.50	-5.63	-4.36
MID	-6.25	-5.37	-4.41
Delta	0.96	1.06	1.56
Range	1.61	2.43	1.97

GI50 / TGI / LC50 axis: +3 +2 +1 0 -1 -2 -3

National Cancer Institute Developmental Therapeutics Program Mean Graphs	NSC :757925/1	Units :Molar	SSPL :0Y1V	EXP. ID :1106NS60
	Report Date :August 02, 2011		Test Date :June 06, 2011	

Panel/Cell Line	$Log_{10}GI50$	$Log_{10}TGI$	$Log_{10}LC50$
**Leukemia**			
CCRF-CEM	-6.41	-5.31	> -4.00
HL-60(TB)	-6.43	-5.74	> -4.00
K-562	-6.43	-5.54	> -4.00
MOLT-4	-6.40	-5.28	> -4.00
RPMI-8226	-6.31	-5.48	> -4.00
SR	-6.53	-6.03	> -4.00
**Non-Small Cell Lung Cancer**			
A549/ATCC	-5.62	-4.62	> -4.00
EKVX	-5.49	-4.60	> -4.00
HOP-62	-5.95	-5.09	-4.26
HOP-92	-5.56	-5.09	> -4.00
NCI-H226	-5.56	-5.01	> -4.00
NCI-H23	-5.93	-5.20	> -4.00
NCI-H322M	-5.66	-4.94	> -4.00
NCI-H460	-6.21	-4.97	> -4.00
NCI-H522	-6.58	-5.86	> -4.00
**Colon Cancer**			
COLO 205	-5.72	-5.47	-5.21
HCC-2998	-5.65	-5.25	-4.45
HCT-116	-6.39	-4.98	-4.34
HCT-15	-6.24	-4.75	> -4.00
HT29	-5.60		> -4.00
KM12	-6.35	-5.14	> -4.00
SW-620	-6.26	-4.99	-4.13
**CNS Cancer**			
SF-268	-6.20	-5.13	> -4.00
SF-295	-5.58	-5.01	> -4.00
SF-539	-6.39	-5.49	-4.05
SNB-19	-5.93	-4.52	> -4.00
SNB-75	-6.10	-5.29	-4.17
U251	-6.18	-4.96	> -4.00
**Melanoma**			
LOX IMVI	-6.31	-5.33	> -4.00
MALME-3M	-6.12	-5.06	> -4.00
M14	-6.36	-5.10	> -4.00
MDA-MB-435	-6.64	-6.24	> -4.00
SK-MEL-2	-6.25	-5.47	> -4.00
SK-MEL-28	-6.01	-5.24	-4.06
SK-MEL-5	-6.40	-5.66	-4.63
UACC-257	-5.72	-5.03	> -4.00
UACC-62	-5.98	-5.40	> -4.00
**Ovarian Cancer**			
IGROV1	-5.59	-4.66	> -4.00
OVCAR-3	-6.35	-5.56	> -4.00
OVCAR-4	-5.77	-5.14	> -4.00
OVCAR-8	-5.91	-5.34	> -4.00
NCI/ADR-RES	-6.31	-4.58	> -4.00
SK-OV-3	-6.13	-5.41	-4.50
**Renal Cancer**			
786-0	-6.14	-5.09	-4.14
A498	-5.61	-5.22	-4.69
CAKI-1	-6.05	-4.39	> -4.00
RXF 393	-6.39	-5.76	
SN12C	-6.05	-4.33	> -4.00
TK-10	-5.54	-4.91	> -4.00
UO-31	-6.28	-5.46	> -4.00
**Prostate Cancer**			
PC-3	-5.86	-5.06	> -4.00
DU-145	-5.71	-4.96	-4.41
**Breast Cancer**			
MCF7	-6.39	-5.25	-4.21
MDA-MB-231/ATCC	-5.59	> -4.00	> -4.00
HS 578T	-6.16	-5.21	> -4.00
BT-549	-6.02	-5.41	> -4.00
T-47D	-5.69	-5.15	-4.32
MDA-MB-468	-5.93	-5.35	> -4.00
MID	-6.05	-5.17	-4.1
Delta	0.59	1.07	1.11
Range	1.15	2.24	1.21

GI50

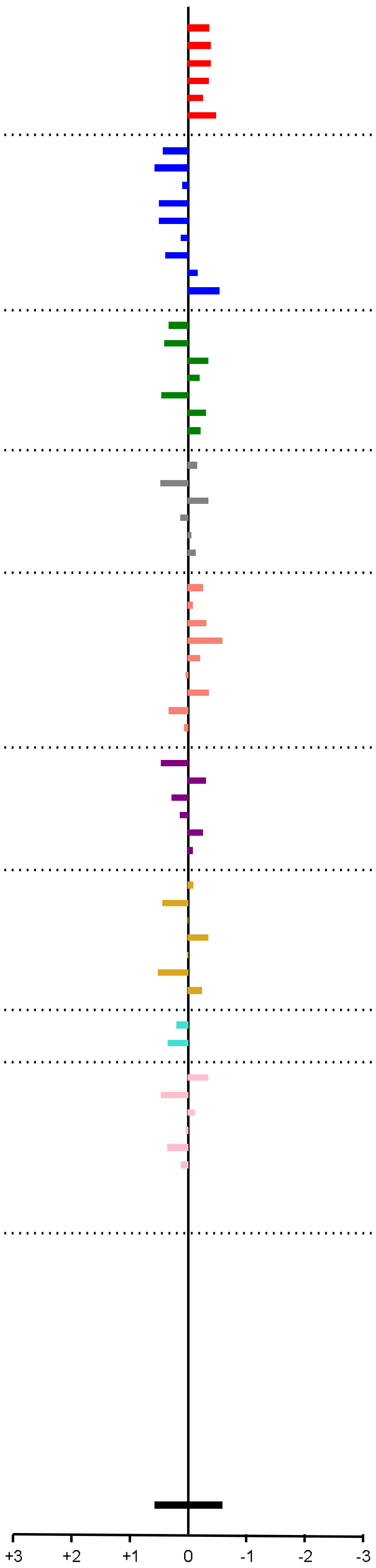

TGI

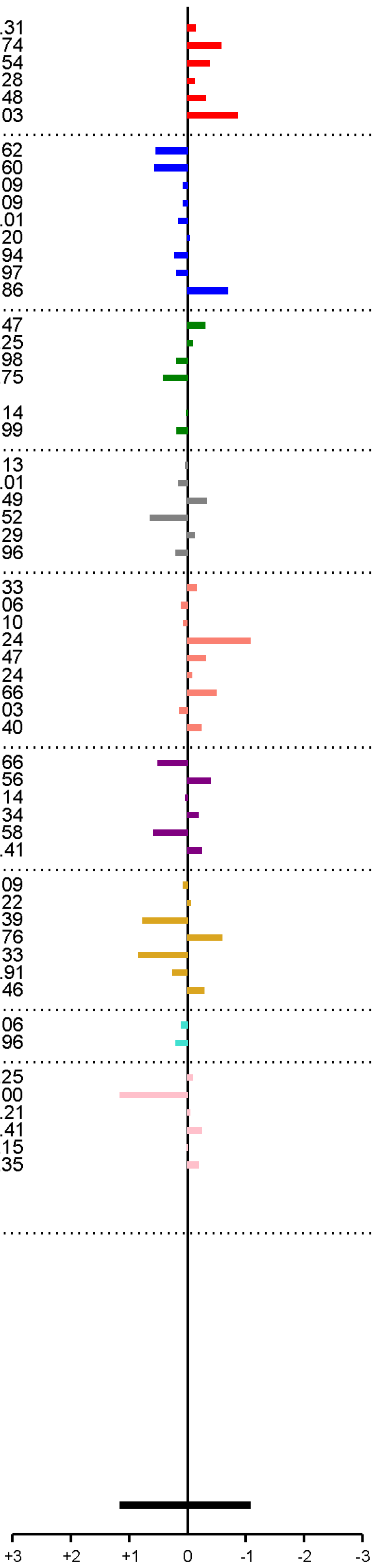

LC50

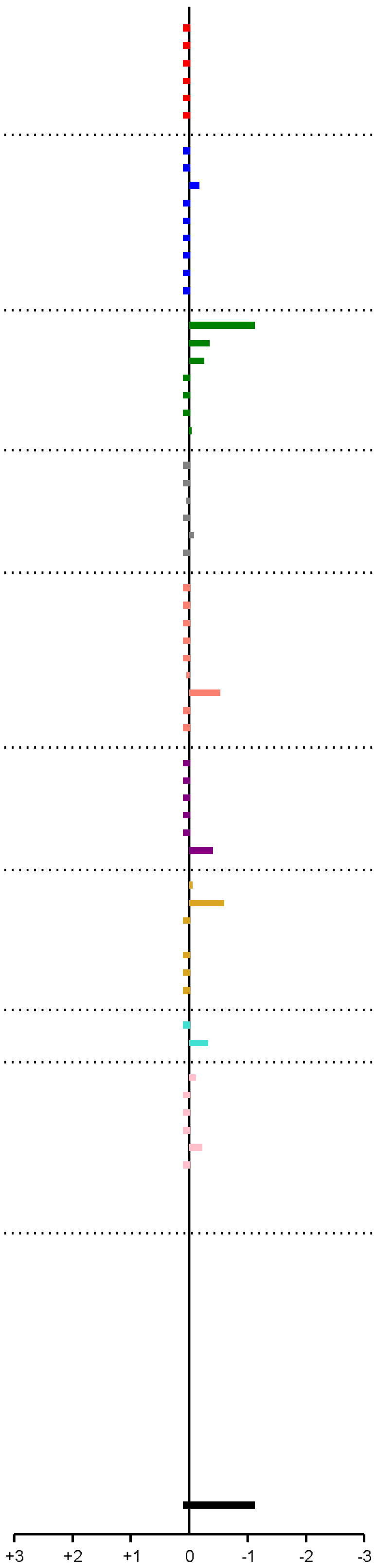

National Cancer Institute Developmental Therapeutics Program Mean Graphs	NSC :757927/1	Units :Molar	SSPL :0Y1V	EXP. ID :1106NS60
	Report Date :August 02, 2011		Test Date :June 06, 2011	

Panel/Cell Line	Log_{10}GI50	Log_{10}TGI	Log_{10}LC50
**Leukemia**			
CCRF-CEM	-6.56		> -4.00
HL-60(TB)	-6.51	-6.06	> -4.00
K-562	-6.54	-5.84	> -4.00
MOLT-4	-6.55	-5.85	> -4.00
RPMI-8226	-6.44	-5.76	> -4.00
SR	-7.36	-6.53	> -4.00
**Non-Small Cell Lung Cancer**			
A549/ATCC	-6.42	-5.47	> -4.00
EKVX	-5.84	-5.06	> -4.00
HOP-62	-6.38	-5.82	-5.40
HOP-92	-6.01	-5.18	> -4.00
NCI-H226	-5.89	-5.46	
NCI-H23	-6.50		> -4.00
NCI-H322M	-6.25	-5.25	> -4.00
NCI-H460	-6.45	-5.96	
NCI-H522	-6.65	-5.99	
**Colon Cancer**			
COLO 205	-5.76	-5.49	-5.23
HCC-2998	-6.23	-5.54	-4.83
HCT-116	-6.45		
HCT-15	-6.42	-5.56	> -4.00
HT29	-5.88	-5.37	> -4.00
KM12	-6.51	-5.94	-5.39
SW-620	-6.33	-5.81	
**CNS Cancer**			
SF-268	-6.34	-5.69	
SF-295	-6.26	-5.59	> -4.00
SF-539	-6.34	-5.72	
SNB-19	-6.17	-5.67	
SNB-75	-6.42	-5.87	
U251	-6.41	-5.82	
**Melanoma**			
LOX IMVI	-6.46		
MALME-3M	-6.21	-5.65	
M14	-6.38	-5.85	
MDA-MB-435	-7.10	-6.41	
SK-MEL-2	-6.27	-5.66	
SK-MEL-28	-6.11	-5.45	-4.48
SK-MEL-5	-6.46	-5.89	
UACC-257	-5.84	-5.21	> -4.00
UACC-62	-6.32	-5.62	> -4.00
**Ovarian Cancer**			
IGROV1	-6.28		> -4.00
OVCAR-3	-6.55	-6.19	-4.52
OVCAR-4	-6.23	-5.25	-4.08
OVCAR-8	-6.34	-5.62	> -4.00
NCI/ADR-RES	-6.53		> -4.00
SK-OV-3	-6.51	-5.91	-5.08
**Renal Cancer**			
786-0	-6.35	-5.82	-5.40
A498	-6.21	-5.71	-5.31
CAKI-1	-6.42	-5.12	> -4.00
RXF 393	-6.64	-6.11	-5.46
SN12C	-6.50		> -4.00
TK-10	-6.19	-5.47	-4.29
UO-31	-6.46	-5.84	
**Prostate Cancer**			
PC-3	-6.30		> -4.00
DU-145	-6.33	-5.80	-5.36
**Breast Cancer**			
MCF7	-6.39	-5.78	
MDA-MB-231/ATCC	-6.13		> -4.00
HS 578T	-6.46	-5.56	> -4.00
BT-549	-6.33	-5.74	
T-47D	-6.39	-5.73	-5.21
MDA-MB-468	-6.63	-6.12	-5.08
_MID	-6.37	-5.71	-4.39
Delta	0.99	0.82	1.07
Range	1.6	1.47	1.46

National Cancer Institute Developmental Therapeutics Program<br>Mean Graphs	NSC :763373/1	Units :Molar	SSPL :0Y1V	EXP. ID :1202NS13
	Report Date :April 25, 2012		Test Date :February 21, 2012	

Panel/Cell Line	Log_{10}GI50	Log_{10}TGI	Log_{10}LC50
**Leukemia**			
CCRF-CEM	-6.57		> -4.00
HL-60(TB)	-6.58	-6.10	> -4.00
K-562	-6.49	-5.81	> -4.00
MOLT-4	-6.56	-5.97	> -4.00
RPMI-8226	-6.60	-6.19	
SR	-7.57	-6.82	> -4.00
**Non-Small Cell Lung Cancer**			
A549/ATCC	-6.38	-5.43	-4.35
EKVX	-6.00	-5.30	> -4.00
HOP-62	-6.13	-5.66	-5.26
HOP-92	-6.21	-5.49	-4.20
NCI-H226	-5.99	-5.35	> -4.00
NCI-H23	-6.42		> -4.00
NCI-H322M	-6.30	-5.33	-4.07
NCI-H460	-6.48	-5.84	-4.39
NCI-H522	-6.76	-6.22	-5.32
**Colon Cancer**			
COLO 205	-5.74	-5.48	-5.23
HCC-2998	-5.65	-5.03	-4.42
HCT-116	-6.57	-5.95	> -4.00
HCT-15	-6.40	-4.96	> -4.00
HT29	-5.83	-5.43	-5.03
KM12	-6.52	-5.82	-4.86
SW-620	-6.32	-5.45	-4.19
**CNS Cancer**			
SF-268	-6.30	-5.29	-4.12
SF-295	-5.70	-5.10	> -4.00
SF-539	-6.26	-5.44	> -4.00
SNB-19	-5.99	-5.06	-4.43
SNB-75	-6.51	-5.99	-5.26
U251	-6.35	-5.44	-4.14
**Melanoma**			
LOX IMVI	-6.35	-5.72	
MALME-3M	-5.98	-5.44	> -4.00
M14	-6.58	-5.95	-5.24
MDA-MB-435	-7.13	-6.45	> -4.00
SK-MEL-28	-6.16	-5.23	-4.49
SK-MEL-5	-6.53	-5.91	-5.41
UACC-257	-6.09	-5.35	-4.32
UACC-62	-6.34	-5.65	
**Ovarian Cancer**			
IGROV1	-6.25	-5.50	> -4.00
OVCAR-3	-6.46	-5.84	-5.03
OVCAR-4	-6.16	-5.23	-4.27
OVCAR-5	-5.57	-5.17	> -4.00
OVCAR-8	-6.33	-5.58	-4.23
NCI/ADR-RES	-6.40		> -4.00
SK-OV-3	-6.42	-5.84	-5.23
**Renal Cancer**			
786-0	-6.42	-5.71	-5.19
A498	-5.92	-5.36	-4.74
ACHN	-6.39	-4.92	> -4.00
CAKI-1	-6.41	-5.33	> -4.00
RXF 393	-6.46	-5.95	-5.35
SN12C	-6.35	> -4.00	> -4.00
TK-10	-6.21	-5.36	-4.46
UO-31	-6.59	-5.84	> -4.00
**Prostate Cancer**			
PC-3	-6.44	-5.73	-4.47
DU-145	-6.38	-5.68	-4.89
**Breast Cancer**			
MCF7	-6.39	-5.67	-4.71
MDA-MB-231/ATCC	-5.92	-4.57	> -4.00
HS 578T	-6.56	-6.16	> -4.00
BT-549	-6.51	-5.63	> -4.00
T-47D	-6.00	-5.60	-5.20
_MID	-6.33	-5.57	-4.41
Delta	1.24	1.25	1
Range	2.0	2.82	1.41

GI50

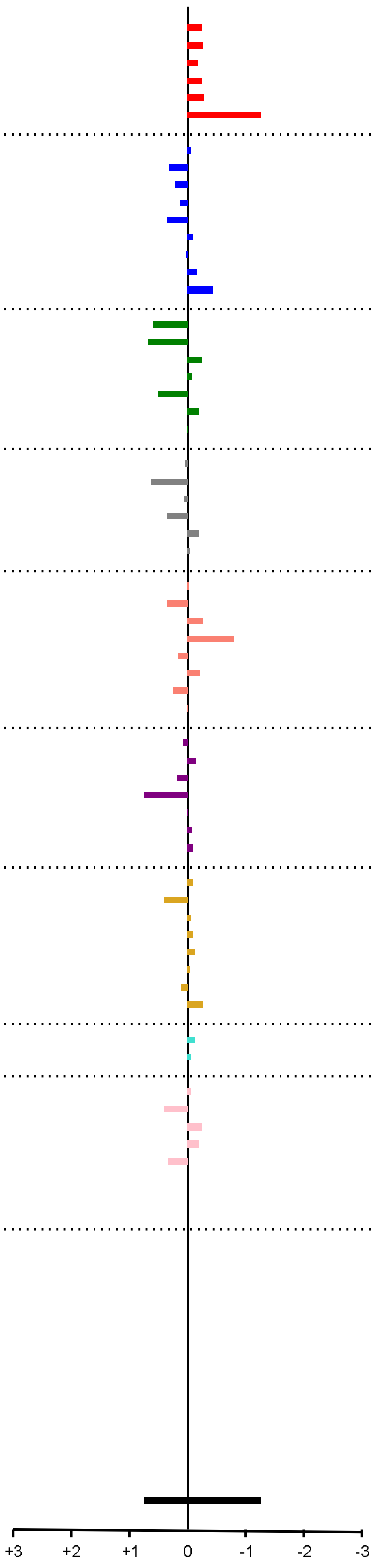

TGI

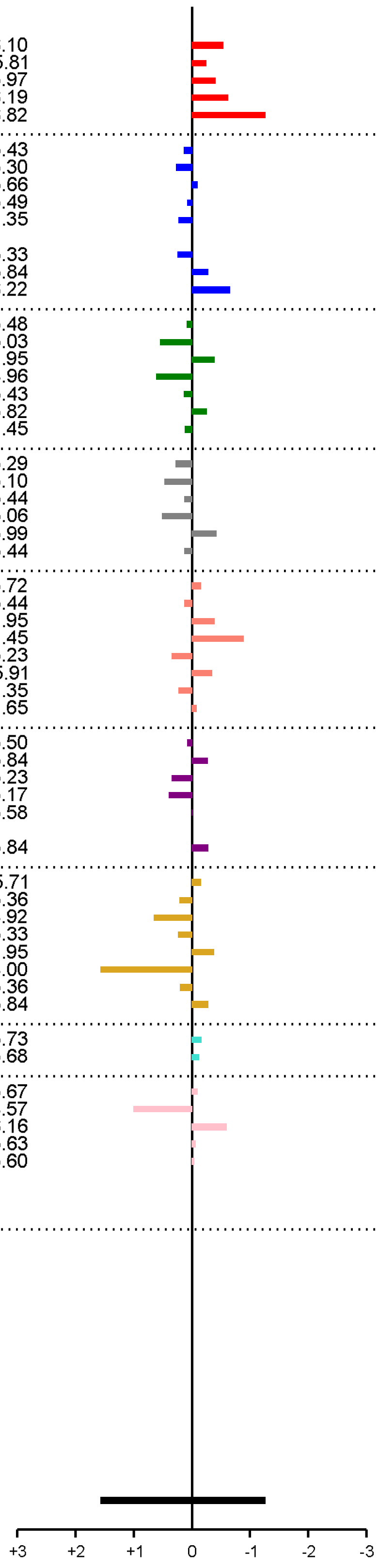

LC50

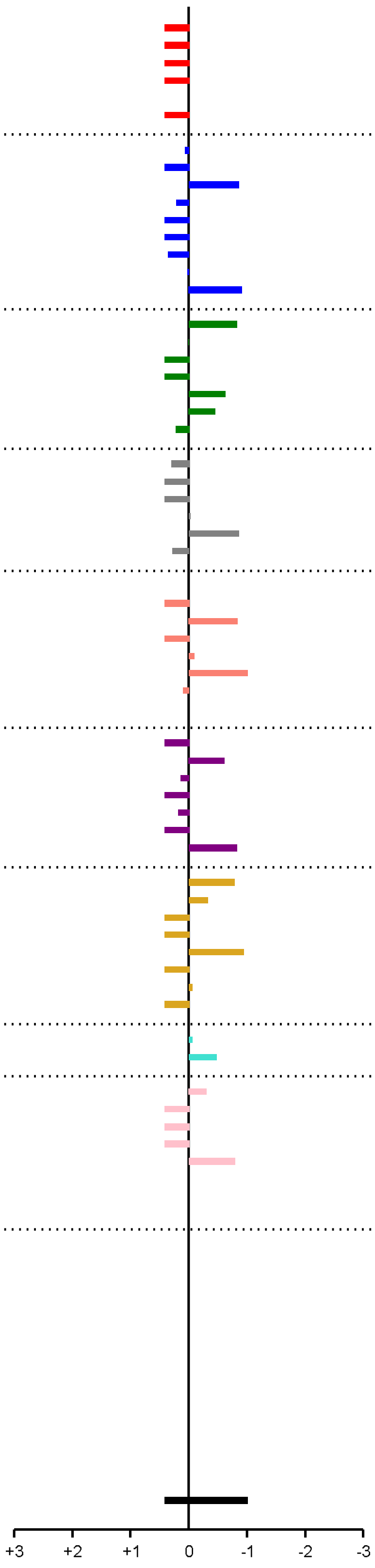

National Cancer Institute Developmental Therapeutics Program
Mean Graphs

NSC :757928/1 | Units :Molar | SSPL :0Y1V | EXP. ID :1106NS60

Report Date :August 02, 2011 | Test Date :June 06, 2011

Panel/Cell Line	Log_{10}GI50	Log_{10}TGI	Log_{10}LC50
**Leukemia**			
CCRF-CEM	-6.08	-5.21	> -4.00
HL-60(TB)	-5.68	-5.22	> -4.00
K-562	-5.84	> -4.00	> -4.00
MOLT-4	-5.91	> -4.00	> -4.00
RPMI-8226	-5.69	-5.22	> -4.00
SR	-6.47		> -4.00
**Non-Small Cell Lung Cancer**			
A549/ATCC	-5.52	-4.34	> -4.00
EKVX	-5.33	> -4.00	> -4.00
HOP-62	-5.47	-4.79	> -4.00
HOP-92	-5.42	-5.02	> -4.00
NCI-H226	-5.48	-4.25	> -4.00
NCI-H23	-5.57	-4.77	> -4.00
NCI-H322M	-5.46	-4.40	> -4.00
NCI-H460	-5.52	> -4.00	> -4.00
NCI-H522	-5.86	-5.20	> -4.00
**Colon Cancer**			
COLO 205	-5.64	-5.33	-5.03
HCC-2998	-5.61	-5.20	> -4.00
HCT-116	-5.51	> -4.00	> -4.00
HCT-15	-5.55	> -4.00	> -4.00
HT29	-5.62		> -4.00
KM12	-5.65	> -4.00	> -4.00
SW-620	-5.44	> -4.00	> -4.00
**CNS Cancer**			
SF-268	-5.61	> -4.00	> -4.00
SF-295	-5.51	-4.46	> -4.00
SF-539	-5.59		> -4.00
SNB-19	-5.57	> -4.00	> -4.00
SNB-75	-5.55	-4.81	> -4.00
U251	-5.58	> -4.00	> -4.00
**Melanoma**			
LOX IMVI	-5.61		> -4.00
MALME-3M	-5.49	-4.36	> -4.00
M14	-5.51	> -4.00	> -4.00
MDA-MB-435	-6.16	-5.09	> -4.00
SK-MEL-2	-5.63	-5.23	> -4.00
SK-MEL-28	-5.59	-5.06	> -4.00
SK-MEL-5	-5.59	-4.88	> -4.00
UACC-257	-5.47	-4.40	> -4.00
UACC-62	-5.58	-5.06	> -4.00
**Ovarian Cancer**			
IGROV1	-5.39	> -4.00	> -4.00
OVCAR-3	-5.60		> -4.00
OVCAR-4	-5.44	-4.64	> -4.00
OVCAR-8	-5.56	-4.91	> -4.00
NCI/ADR-RES	-5.43	> -4.00	> -4.00
SK-OV-3	-5.65	-5.28	> -4.00
**Renal Cancer**			
786-0	-5.50	> -4.00	> -4.00
A498	-5.52	-5.06	> -4.00
CAKI-1	-5.66	> -4.00	> -4.00
RXF 393	-5.78	-5.40	
SN12C	-5.65	> -4.00	> -4.00
TK-10	-5.41	-4.48	> -4.00
UO-31	-5.63	-4.47	> -4.00
**Prostate Cancer**			
PC-3	-5.52	-4.17	> -4.00
DU-145	-5.48	-4.46	> -4.00
**Breast Cancer**			
MCF7	-5.52	-4.89	> -4.00
MDA-MB-231/ATCC	-5.44	> -4.00	> -4.00
HS 578T	-5.56	-4.31	> -4.00
BT-549	-5.67	-5.11	> -4.00
T-47D	-5.53	-4.88	-4.02
MDA-MB-468	-5.65	-5.24	> -4.00
_MID	-5.6	-4.56	-4.02
Delta	0.87	0.84	1.01
Range	1.14	1.4	1.03

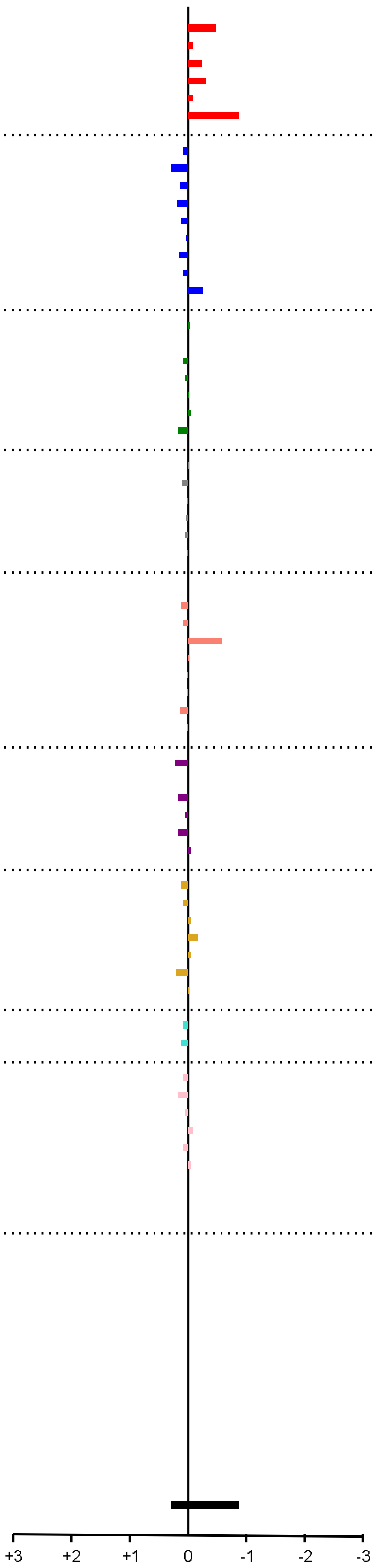

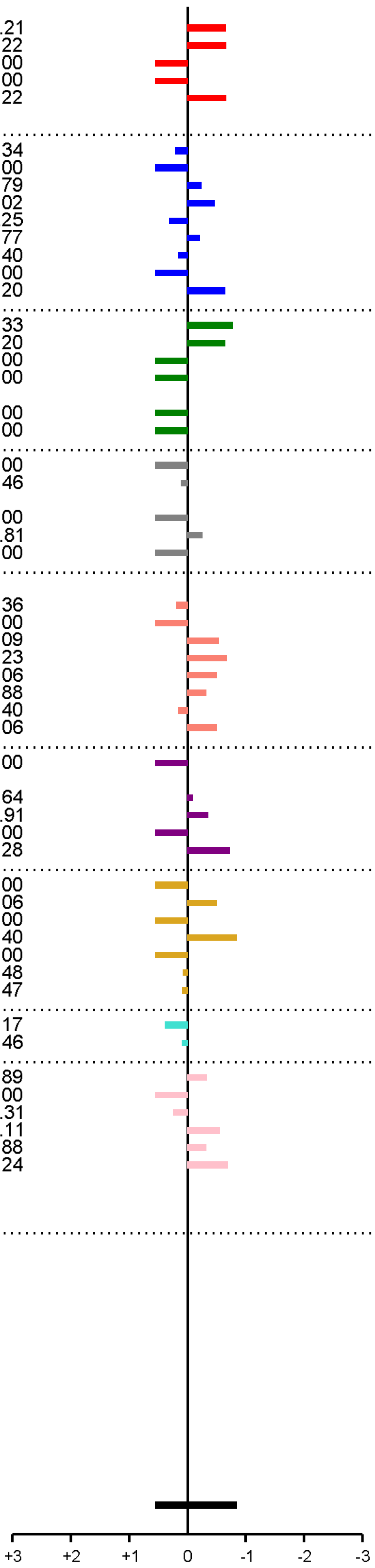

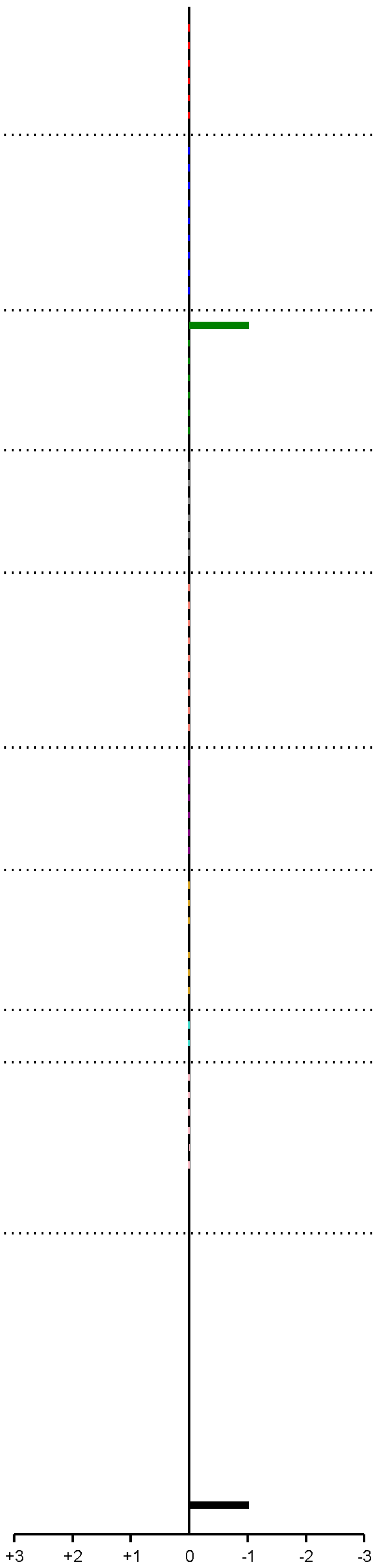

National Cancer Institute Developmental Therapeutics Program<br>Mean Graphs	NSC :763374/1	Units :Molar	SSPL :0Y1V	EXP. ID :1202NS13
	Report Date :April 25, 2012		Test Date :February 21, 2012	

Panel/Cell Line	Log_{10}GI50	Log_{10}TGI	Log_{10}LC50
**Leukemia**			
CCRF-CEM	-5.68	-5.21	> -4.00
HL-60(TB)	-5.43	-4.92	> -4.00
K-562	-5.44	-4.45	> -4.00
MOLT-4	-5.59	-4.93	> -4.00
RPMI-8226	-5.68	-5.25	> -4.00
SR	-5.72	-5.21	> -4.00
**Non-Small Cell Lung Cancer**			
A549/ATCC	-5.28	-4.56	> -4.00
EKVX	-5.41	-4.14	> -4.00
HOP-62	-5.39	-4.76	-4.09
HOP-92	-5.64	-5.03	> -4.00
NCI-H226	-5.46	-4.65	> -4.00
NCI-H23	-5.63		> -4.00
NCI-H322M	-5.33	-4.09	> -4.00
NCI-H460	-5.51	-4.73	> -4.00
NCI-H522	-5.59	-5.10	> -4.00
**Colon Cancer**			
COLO 205	-5.69	-5.40	-5.10
HCC-2998	-5.45	-4.85	-4.03
HCT-116	-5.61	-4.91	> -4.00
HCT-15	-5.26	> -4.00	> -4.00
HT29	-5.89	-5.57	-5.26
KM12	-5.46	-4.85	> -4.00
SW-620	-5.50	-4.78	> -4.00
**CNS Cancer**			
SF-268	-5.34	> -4.00	> -4.00
SF-295	-5.56	-5.01	> -4.00
SF-539	-5.41	> -4.00	> -4.00
SNB-19	-5.47	> -4.00	> -4.00
SNB-75	-5.77	-4.92	> -4.00
U251	-5.50	-4.87	-4.18
**Melanoma**			
LOX IMVI	-5.50	> -4.00	> -4.00
MALME-3M	-5.41	> -4.00	> -4.00
M14	-5.51	-4.81	> -4.00
MDA-MB-435	-5.57	-4.74	> -4.00
SK-MEL-2	-5.54	-5.17	> -4.00
SK-MEL-28	-5.52	-4.88	-4.04
SK-MEL-5	-5.43	-4.44	> -4.00
UACC-257	-5.47	-4.75	> -4.00
UACC-62	-5.47	-4.36	> -4.00
**Ovarian Cancer**			
IGROV1	-5.36	> -4.00	> -4.00
OVCAR-3	-5.42	-4.48	> -4.00
OVCAR-4	-5.61	-4.77	> -4.00
OVCAR-5	-5.00	-4.55	-4.10
OVCAR-8	-5.52	-4.90	> -4.00
NCI/ADR-RES	-4.78	> -4.00	> -4.00
SK-OV-3	-5.63	-5.31	> -4.00
**Renal Cancer**			
786-0	-5.56	-4.48	> -4.00
A498	-5.51	-4.60	> -4.00
ACHN	-5.05	> -4.00	> -4.00
CAKI-1	-5.47	> -4.00	> -4.00
RXF 393	-5.65	-5.31	-4.56
SN12C	-5.36	> -4.00	> -4.00
TK-10	-5.21	-4.44	> -4.00
UO-31	-5.43	-4.23	> -4.00
**Prostate Cancer**			
PC-3	-5.49	-4.73	> -4.00
DU-145	-5.31	-4.45	> -4.00
**Breast Cancer**			
MCF7	-5.53	-4.94	-4.18
MDA-MB-231/ATCC	-5.45	> -4.00	> -4.00
HS 578T	-5.74	-5.17	> -4.00
BT-549	-5.69	-5.09	> -4.00
T-47D	-5.19	-4.43	> -4.00

	GI50	TGI	LC50
_MID	-5.48	-4.64	-4.06
Delta	0.41	0.93	1.2
Range	1.11	1.57	1.26

GI50

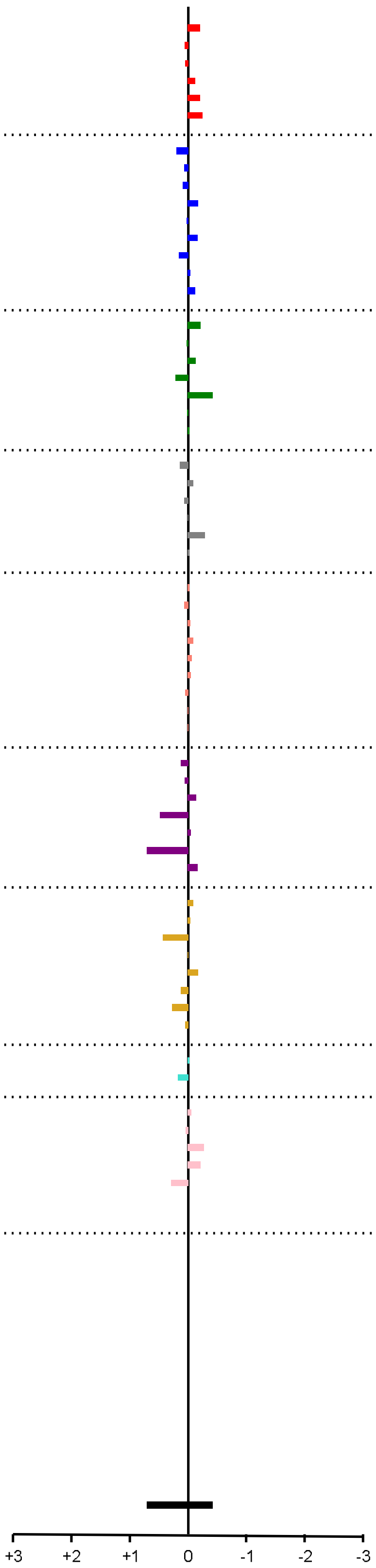

TGI

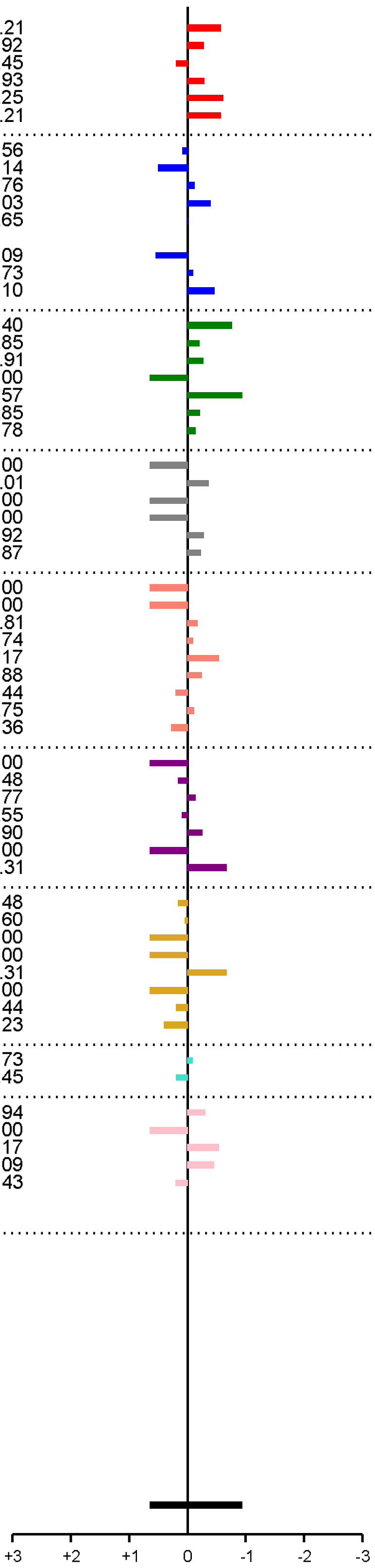

LC50

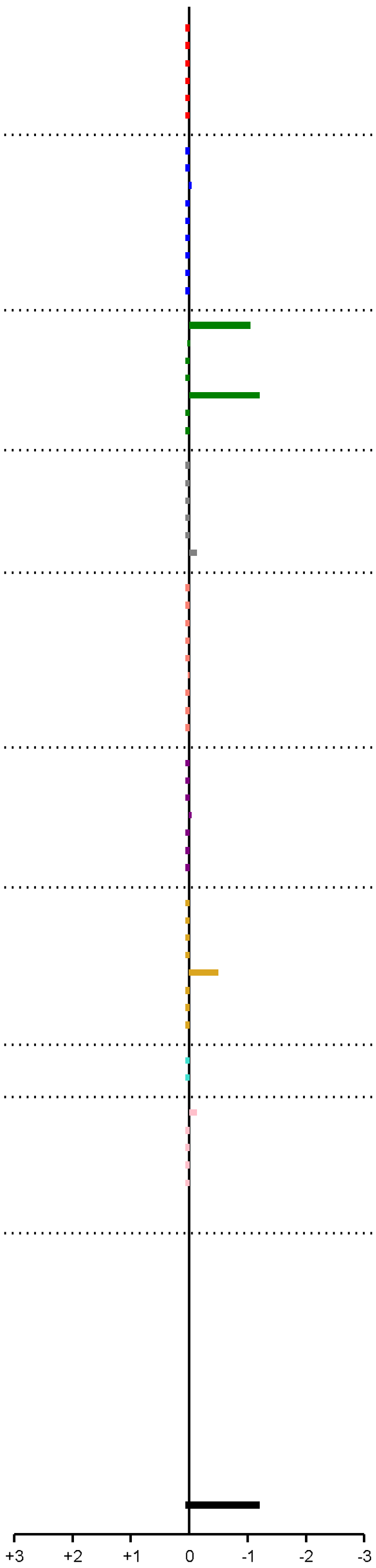

Supplement: Supplementary file 1 — All the structures were drawn by using Maestro. Their structures were minimized using Macromodel minimization panel using the OPLS-2005 force field and GB/SA water model with a constant dielectric of 1.0 Polak-Ribiere frst derivative, conjugate gradient minimization was employed with maximum iterations of 1000 and convergence threshold of a gradient to < 0.05 kJ/Å-mol. LigPrep2.0 module of Schrodinger was used to generate possible ionization states at target pH 7.0±2.0. All possible tautomeric states at this pH were also generated using the tautomerizer module of LigPrep2.0. The resulting structures were saved in ∗.mae format for further experiments. [file 239354.f1.pdf]
